# Invariant patterns of clonal succession determine specific clinical features of myelodysplastic syndromes

**DOI:** 10.1038/s41467-019-13001-y

**Published:** 2019-11-26

**Authors:** Yasunobu Nagata, Hideki Makishima, Cassandra M. Kerr, Bartlomiej P. Przychodzen, Mai Aly, Abhinav Goyal, Hassan Awada, Mohammad Fahad Asad, Teodora Kuzmanovic, Hiromichi Suzuki, Tetsuichi Yoshizato, Kenichi Yoshida, Kenichi Chiba, Hiroko Tanaka, Yuichi Shiraishi, Satoru Miyano, Sudipto Mukherjee, Thomas LaFramboise, Aziz Nazha, Mikkael A. Sekeres, Tomas Radivoyevitch, Torsten Haferlach, Seishi Ogawa, Jaroslaw P. Maciejewski

**Affiliations:** 1 Department of Translational Hematology & Oncology Research, Taussig Cancer Institute, Cleveland Clinic, Cleveland, OH USA; 2 Department of Pathology and Tumor Biology, Graduate School of Medicine, Kyoto University, Kyoto, Japan; 3 Laboratory of DNA Information Analysis, Human Genome Center, Institute of Medical Science, The University of Tokyo, Tokyo, Japan; 4 Department of Hematology and Medical Oncology, Taussig Cancer Institute, Cleveland Clinic, Cleveland, OH USA; 5 Department ofGenetics and Genome Sciences, Case Western Reserve University, Cleveland, OH USA; 6 MLL Munich Leukemia Laboratory, Munich, Germany

**Keywords:** Myelodysplastic syndrome, Cancer genomics

## Abstract

Myelodysplastic syndromes (MDS) arise in older adults through stepwise acquisitions of multiple somatic mutations. Here, analyzing 1809 MDS patients, we infer clonal architecture by using a stringent, the single-cell sequencing validated PyClone bioanalytic pipeline, and assess the position of the mutations within the clonal architecture. All 3,971 mutations are grouped based on their rank in the deduced clonal hierarchy (dominant and secondary). We evaluated how they affect the resultant morphology, progression, survival and response to therapies. Mutations of *SF3B1, U2AF1, and TP53* are more likely to be dominant, those of *ASXL1, CBL, and KRAS* are secondary. Among distinct combinations of dominant/secondary mutations we identified 37 significant relationships, of which 12 affect clinical phenotypes, 5 cooperatively associate with poor prognosis. They also predict response to hypomethylating therapies. The clonal hierarchy has distinct ranking and the resultant invariant combinations of dominant/secondary mutations yield novel insights into the specific clinical phenotype of MDS.

## Abstract (web summary)

Stepwise acquisition of mutations gives rise to myelodysplastic syndrome (MDS) in older adults. Here, the authors infer the clonal hierarchy of 1809 MDS patients, revealing insights into the evolution of dominant/secondary mutations and how these impact clinical phenotypes like leukemic progression and therapy response.

## Introduction

Myelodysplastic syndromes (MDS) are a clinically and molecularly heterogeneous collections of diseases^1,2^. They affect older adults and are characterized by dysplastic hematopoiesis, cytopenias, and a propensity for progression to acute myeloid leukemia (AML)^3–6^. Recent discoveries enabled by next-generation sequencing (NGS) have led to new insights into the pathogenic origins of MDS^7–16^.

Irrespective of origins, MDS pathogenesis includes initial ancestral lesion followed by the stepwise acquisition of subsequent somatic mutations resulting in a highly diverse clonal hierarchy^17^. While strong driver hits result in MDS instantly in form of a de novo disease, some of the founder mutations in MDS originate from subclinical clonal expansions, referred to as clonal hematopoiesis (CH) present in the blood of some otherwise healthy individuals^18,19^. The prevalence of CH increases with age and is highly suggestive of a prodromal stage of MDS, i.e., CH may be a pre-MDS state. In some individuals, CH progresses to MDS via acquisition of secondary hits. However, due to competing risks of mortality and long latencies of subsequent transforming phenotypes, only a fraction of carriers with CH mutations develops MDS^20–23^. Initially, asymptomatic CH mutations are thus much less penetrant than typical AML hits, e.g. t(15;17) or translocations involving *MLL*^24,25^.

We hypothesized that partitioning patients based on ancestral and secondary hits would yield new metrics of MDS pathogenesis that are predictive of clinical and morphologic features and prognostic of outcomes. Heretofore, mapping mutation-state combinatorics to phenotypes have demonstrated a tremendous complexity, but have not yielded generalizable rules; correlations with classical morphologic subdivisions have been weak^26^. Clustering mutations by their rank in the clonal hierarchy or ancestral hit-deducted derivation as de novo or CH-related disease may illuminate disease teleology and consequent clinical outcome. Fundamental to the interpretation of clonal hierarchy is determining whether ancestral hits are succeeded by random vs. predetermined secondary hits, and whether mutation interactions influence phenotypes. Primary hits may drive general phenotypes that are then modulated by secondary hits or alternatively, they may initiate leukemogenic processes and secondary hits may fully determine phenotypes and progression rates.

Clonal hierarchies are best characterized using deep sequencing on serial samples starting with CH and progressing through MDS to AML. Single-cell sequencing allows for cross-sectional analysis of clonal architecture and most precise recapitulation of clonal ontology^2,27^. Neither of these two approaches is suitable, though, to clinical realities: patients rarely have serial samples obtained, and routine single-cell sequencing of many patients is not yet feasible, certainly not for screenings for CH in healthy or in other large clinical cohorts. While single-cell investigation of individual patients can yield excellent results, broadly investigate this method to appropriately sized cohorts of patients precludes generalizable conclusions commensurate with the individual diversity. Consequently, the Beta Binomial emission model implemented in PyClone has been developed to recapitulate clonal hierarchy^28^. Limitations aside, these approaches provide hierarchical ranks of mutations that reflect clonal succession from primary/dominant hits to subsequent secondary hits.

Here, using a large cohort of patients and innovative analytical approaches, we investigated the origins of MDS and characterize the extent to which clonal succession rules exist and are predictive of MDS morphologic features and prognoses.

## Results

### Mutational profile of MDS

**Fig. 1:**
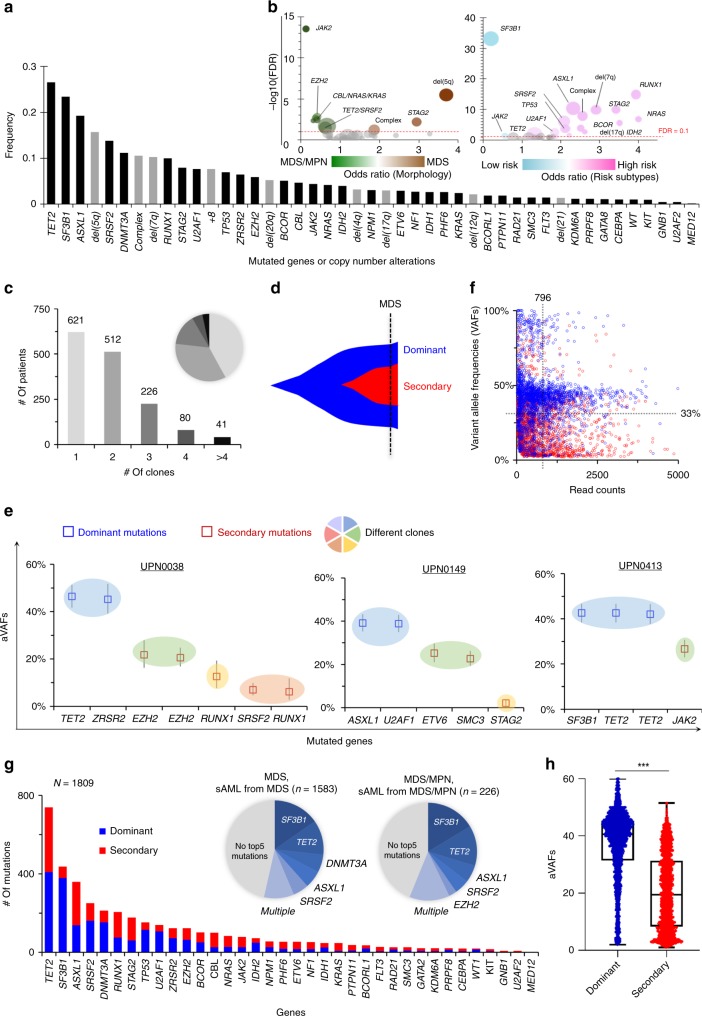
Mutational landscapes, and dominant/secondary mutations. **a** Frequency of the 36 mutated genes (dark bars) and copy-number alterations (gray bars). **b** Clinical features associated with genetic hits. Effect of frequent genetic hits (>2%, *n* = 30) were assessed. Bubble sizes encode the frequency of mutations in 1809 patients; odds ratios (OR) of MDS (*n* = 1583) vs. MDS/MPN (*n* = 226) and low- (*n* = 1043) vs. high risk (*n* = 766) are plotted on the *x*-axes. Negative log10 false discovery rates (FDR, i.e., *q*-values) are shown on the *y*-axes; our FDR cutoff of interest, 0.1, is shown as a dashed horizontal red line. **c** The recent advanced algorithm called PyClone allowed the inference of clonal structure. All 3971 mutations, identified in 1809 samples, were evaluated in each sample. Multiple clones were found in 859 samples. The bar graphs and pie chart show the number and the fraction of samples with different number of clones, respectively. **d** Clonal evolution of MDS. Ancestral/initial hits were classified dominant, and subsequent/secondary hits were classified as secondary mutations. **e** Representative three samples with multiple clones. Copy-number-adjusted variant allele frequencies (aVAFs) (*y*-axis) and mutated genes (*x*-axis) in three illustrative samples are shown. Blue and red squares depict dominant and secondary mutations. Circles with different colors show the different clones estimated by PyClone^28^. **f** Sequencing read counts and raw VAFs. This scatter plot depicts 3971 mutations identified in 1809 patients. Blue and red bars indicate dominant and secondary mutations, respectively. Dotted vertical and horizontal lines depict mean total read counts and VAFs, respectively. **g** Distribution of dominant, co-dominant, and secondary mutations in all 36 genes in our panel. Dominant (*n* = 2155) and secondary mutations (*n* = 1816) are shown. Pie charts show the fraction of dominant mutations for each gene. The top five most frequently mutated genes are indicated in the left (MDS, sAML from MDS) and right (MDS/MPN, sAML form MDS/MPN), respectively. **h** Distribution of aVAFs between dominant vs. secondary mutations. Dot plots depict dominant (blue) and secondary (red) mutations. Box and whiskers indicate median and minimum to maximum of aVAFs for dominant and secondary mutations. ***P < 0.0001 (Mann–Whitney’s *U* test).

Our study included newly diagnosed and fully annotated 1809 patients with MDS or MDS/MPN overlap, including lower- and higher-risk subtypes (LR or HR; Table 1 also see description of the patients in the Methods section) analyzed with clinical parameters and a panel of the most frequently mutated 36 myeloid genes using deep targeted NGS (TS) (Supplementary Table 1). In a subset (12%, *n* = 225/1809) of these patients, whole-exome sequencing (WES) was performed to assess the congruence of the results with targeted sequencing (Supplementary Table 2). A total of 1169 patients from a previous report^29^ (52% of the previous cohort^29^) were fully annotated by copy-number alterations, read counts of mutations, and uniformly applied morphologic assessment for inferring accurate clonal structure, which were included in this study. We also added 640 entirely new patients (Supplementary Fig. 1). Among 36 genes tested after removing SNPs/errors, 3971 somatic mutations were recorded and combined with copy-number alterations (CNA; Supplementary Figs. 2–5). The most frequently mutated genes/CNAs were *TET2* (27%), *SF3B1* (23%), *ASXL1* (19%), del(5q) (16%), *SRSF2* (14%), *DNMT3A* (11%), and −7/del7q (10%), each present in >10% of patients (Fig. 1a). Overall, 30 genetic hits (mutations/CNAs) were identified in >2% of patients. We then examined the pairing likelihood of genomic lesions: 110 of the most common significantly associated mutant combinations were found (Supplementary Fig. 6 and Supplementary Data 1). To assess the impact of mutations on the phenotype, we then ranked them in two-dimensional space according to the odds of MDS vs. MDS/MPN association (history of MDS or MPN/MDS for the corresponding sAML) or LR vs. HR subtypes. For instance, del(5q), *STAG2* mutations, and complex karyotype more likely associated with MDS features (Fig. 1b), while *JAK2, EZH2*, and RAS pathway (*NRAS/KRAS/CBL*) mutations associate with MDS/MPN overlap features. Similarly, *RUNX1, STAG2* mutations, or -7/del(7q) were more likely to group with HR subtypes in contrast to *SF3B1*, and *JAK2* mutations predictive of LR subtypes.

**Table 1:** Demographic data of 1809 MDS patients subject to sequencing.

Parameter	WES	TS^a^	Total cohort
Patient numbers	225	1584	1809
Median age	70.9	71.8	71.8
Ratio of male to female	1.2	1.7	1.6
Survival (median, months)	25	28	27
*Diagnosis*			
MDS	152 (68%)	1294 (82%)	1446 (80%)
MDS/MPN	41 (18%)	171 (11%)	212 (12%)
sAML^b^	32 (14%)	119 (7%)	151 (8%)
*Subtype of progression*			
Low risk^c^	117 (52%)	926 (59%)	1043 (58%)
High risk^c^	108 (48%)	658 (41%)	766 (42%)

*WES* whole-exome sequencing, *TS* targeted sequencing, *MDS* myelodysplastic syndromes, *MPN* myeloproliferative neoplasms, *sAML* secondary acute myeloid leukemia from MDS or MDS/MPN

^a^In total, 36 common genes were sequenced in the entire cohort

^b^All sAML cases were determined derived from MDS (*n* = 137) or MDS/MPM (*n* = 14)

^c^Two groups based on IPSS-R scores (low risk as <=3.5 and high risk as >3.5) according to the transformation risk (Pfeilstöcker, M. et al.^47^)

### Ancestral hits and recapitulation of clonal hierarchy

**Fig. 2:**
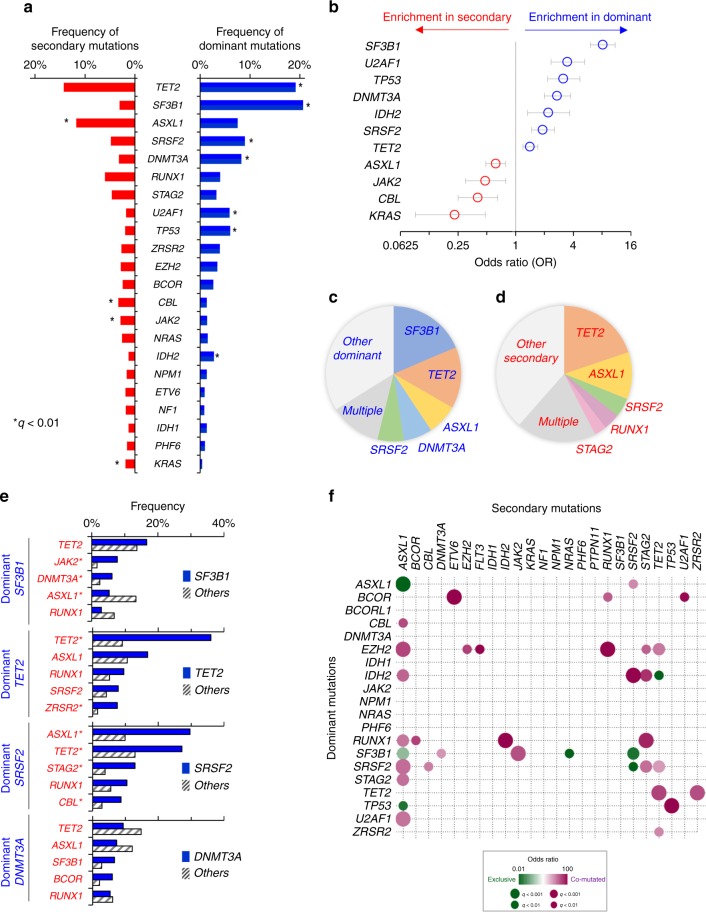
Comparison of dominant- and secondary mutations, and their combinations. **a** Comparison of frequencies of dominant vs. secondary recurrent somatic mutations (recurrent is defined here as >2%, *n* = 22); * indicate *q* < 0.01, where *q* is the Benjamini–Hochberg corrected Fisher’s exact test *P*-value. **b** Odds ratios and 95% confidential intervals of genes more likely to have dominant (blue) or secondary (red) mutations. **c**, **d** Proportion of top five frequent genes for (**c**) patients with dominant mutations, and (**d**) those with secondary mutations. Patients with multiple dominant or secondary mutations in the top five genes belong to the group called Multiple. **e** Nonrandom associations between dominant and secondary mutations. Shown are secondary mutations in each of top four dominant mutational groups. Bars indicate frequencies of secondary mutations in patients with (blue) vs. without (striped) dominant mutation. *q < 0.01, Fisher’s exact test *P-*values with Benjamini–Hochberg correction. **f** Correlation of dominant and secondary mutations. Recurrent dominant (>1%, *n* = 16) and secondary mutations (>1%, *n* = 22) are given in the *y*- and *x*-axes, respectively. Co-occurrence and mutually exclusivity are encoded in purple and green color gradients, respectively. Circle sizes encode *q*-values (Fisher’s exact test *P-*values with Benjamini–Hochberg corrections).

Typically, one of the mutational events initiates the subsequent cascade of subclonal events, resulting in clonal evolution. We hypothesized that most dominant mutations and subsequent hits can be ranked and assessed in such context irrespective of the nominal clonal burden. Thus, the impact of individual mutations may depend on their position within the clonal hierarchy with a special role for the earliest (ancestral or dominant) vs. secondary events. To identify significant differences between them, a stringent bioanalytic pipeline relying on differential clonal size in accordance with the read counts was employed. We investigated the temporal ranking of mutations during clonal evolution by the PyClone pipeline, which has been validated using single-cell sequencing^28^. It uses a Bayesian clustering method for grouping sets of deeply sequenced somatic mutations into putative clonal clusters, which revealed speculated clonal structure in our MDS patients, of which 859 patients harbored multiple (median 2, range 1–7) (Fig. 1c). Clonal hierarchy was reduced to a one-dimensional ordered 2-level feature space of primary/dominant and secondary mutations (Fig. 1d). Mutations belonging to the largest clone were defined as dominant mutations, and other clones as secondary mutations (Supplementary Data 2). Several representative patients are shown (Fig. 1e; Supplementary Fig. 7). Mean variant allele frequencies (VAFs) and total read counts were 33% and 796, respectively (Fig. 1f). Also, dominant clone distribution, as determined by targeted panel, matched that of founder clones by WES, indicating that the targeted panel does reflect the spectrum of most important early mutations (Supplementary Fig. 8). Distribution of dominant and secondary mutations depended on each gene and morphologic features (Fig. 1g). In total, 2155 (54%) band 1816 (46%) mutations were judged to be dominant and secondary, respectively. Median VAFs of dominant and secondary mutations were 40.6 and 19.4% (Mann–Whitney’s *U* test; *P* < 0.0001) (Fig. 1h). Mutations of *SF3B1, U2AF1, TP53, DNMT3A, IDH2*, *SRSF2*, and *TET2* were more likely to be dominant, while those of *ASXL1, JAK2, CBL*, and *KRAS* were more likely to be secondary (Fig. 2a, b). For some genes, canonical vs. other missense or truncating mutations may have a different position within the clonal hierarchy. For instance, *DNMT3A R882* mutations were more likely to be dominant compared with truncating or other missense mutations, which were more likely to be secondary (Supplementary Fig. 9). The top five dominant mutation genes were *SF3B1, TET2, ASXL1, DNMT3A*, and *SRSF2 (*Fig. 2c). *TET2, ASXL1*, and *SRSF2* were also identified in secondary mutations (Fig. 2d).

### Molecular association of dominant and secondary mutations

Our results suggest that, starting with the initial mutations, subsequent hits are not random, but rather follow a certain order (Fig. 2e), and that certain mutation combinations (dominant/secondary) occur more commonly. Statistically significant correlations were found across 37 distinctive gene pairs (Fig. 2f and Table 2); 30 and 7 mutation pairs either coincided or were mutually exclusive, respectively. For example, *TP53* dominant mutations were more likely to precede secondary *TP53* mutations, but less likely secondary *ASXL1* mutations (Supplementary Fig. 10). Similarly, dominant *IDH2* mutations preceded secondary *ASXL1, SRSF2*, and *STAG2* mutations. Dominant *TET2* mutations also preceded secondary *TET2*, and *ZRSR2* mutations. While *ASXL1* was commonly affected by specific secondary mutations, they occurred preferentially in the context of given dominant alterations. For instance, dominant *SRSF2, U2AF1, EZH2, RUNX1, STAG2, IDH2*, and *CBL* mutations were more likely to precede secondary *ASXL1* mutations, and conversely dominant *TP53*, *ASXL1*, and *SF3B1* mutations were less likely to co-occur secondary *ASXL1* mutations (Supplementary Fig. 11).

**Table 2:** Significantly correlated pairs between dominant and secondary events.

Dominant	Secondary	Co-occurrence	Dominant only	Secondary only	Intact	OR	*P*-value	*q*-value	Co-occurrence (CO) vs. mutually exclusive (ME)
*ASXL1*	*ASXL1*	1	136	211	1461	0.1	6.14E-07	2.77E-05	ME
*ASXL1*	*SRSF2*	16	121	72	1600	2.9	6.20E-04	8.15E-03	CO
*BCOR*	*ETV6*	8	40	27	1734	12.8	1.85E-06	6.99E-05	CO
*BCOR*	*RUNX1*	10	38	99	1662	4.4	3.93E-04	5.72E-03	CO
*BCOR*	*U2AF1*	6	42	26	1735	9.5	1.36E-04	2.63E-03	CO
*CBL*	*ASXL1*	10	15	202	1582	5.2	2.64E-04	4.38E-03	CO
*EZH2*	*ASXL1*	25	38	187	1559	5.5	5.99E-09	4.73E-07	CO
*EZH2*	*EZH2*	8	55	43	1703	5.7	2.69E-04	4.39E-03	CO
*EZH2*	*FLT3*	5	58	13	1733	11.4	2.64E-04	4.38E-03	CO
*EZH2*	*RUNX1*	23	40	86	1660	11.1	1.15E-13	2.17E-11	CO
*EZH2*	*STAG2*	10	53	74	1672	4.3	4.61E-04	6.41E-03	CO
*EZH2*	*TET2*	22	41	236	1510	3.4	2.26E-05	5.38E-04	CO
*IDH2*	*ASXL1*	17	33	195	1564	4.1	2.26E-05	5.38E-04	CO
*IDH2*	*SRSF2*	16	34	72	1687	11.0	3.24E-10	3.41E-08	CO
*IDH2*	*STAG2*	11	39	73	1686	6.5	9.52E-06	2.65E-04	CO
*IDH2*	*TET2*	0	50	258	1501	Infinity	6.99E-04	8.70E-03	ME
*RUNX1*	*ASXL1*	22	51	190	1546	3.5	1.25E-05	3.20E-04	CO
*RUNX1*	*BCOR*	8	65	37	1699	5.6	3.09E-04	4.88E-03	CO
*RUNX1*	*IDH2*	8	65	15	1721	14.1	1.44E-06	5.93E-05	CO
*RUNX1*	*STAG2*	17	56	67	1669	7.5	9.50E-09	6.42E-07	CO
*SF3B1*	*ASXL1*	20	354	192	1243	0.4	5.26E-06	1.72E-04	ME
*SF3B1*	*DNMT3A*	23	351	35	1400	2.6	7.71E-04	9.35E-03	CO
*SF3B1*	*JAK2*	29	345	24	1411	4.9	3.05E-08	1.92E-06	CO
*SF3B1*	*NRAS*	1	373	46	1389	0.1	3.66E-04	5.52E-03	ME
*SF3B1*	*SRSF2*	3	371	85	1350	0.1	4.56E-06	1.54E-04	ME
*SRSF2*	*ASXL1*	48	114	164	1483	3.8	6.05E-11	7.15E-09	CO
*SRSF2*	*CBL*	14	148	47	1600	3.2	6.53E-04	8.41E-03	CO
*SRSF2*	*SRSF2*	0	162	88	1559	Infinity	3.73E-04	5.52E-03	ME
*SRSF2*	*STAG2*	21	141	63	1584	3.7	6.40E-06	1.95E-04	CO
*SRSF2*	*TET2*	44	118	214	1433	2.5	5.46E-06	1.72E-04	CO
*STAG2*	*ASXL1*	19	41	193	1556	3.7	2.33E-05	5.38E-04	CO
*TET2*	*TET2*	125	222	133	1329	5.6	1.00E-31	9.47E-29	CO
*TET2*	*ZRSR2*	26	321	23	1439	5.1	7.51E-08	4.44E-06	CO
*TP53*	*ASXL1*	2	107	210	1490	0.1	1.75E-04	3.18E-03	ME
*TP53*	*TP53*	19	90	17	1683	20.8	5.12E-15	1.21E-12	CO
*U2AF1*	*ASXL1*	32	75	180	1522	3.6	1.27E-07	7.06E-06	CO
*ZRSR2*	*TET2*	23	49	235	1502	3.0	8.69E-05	1.79E-03	CO

### Pairs of dominant and secondary mutations impact phenotypes

**Fig. 3:**
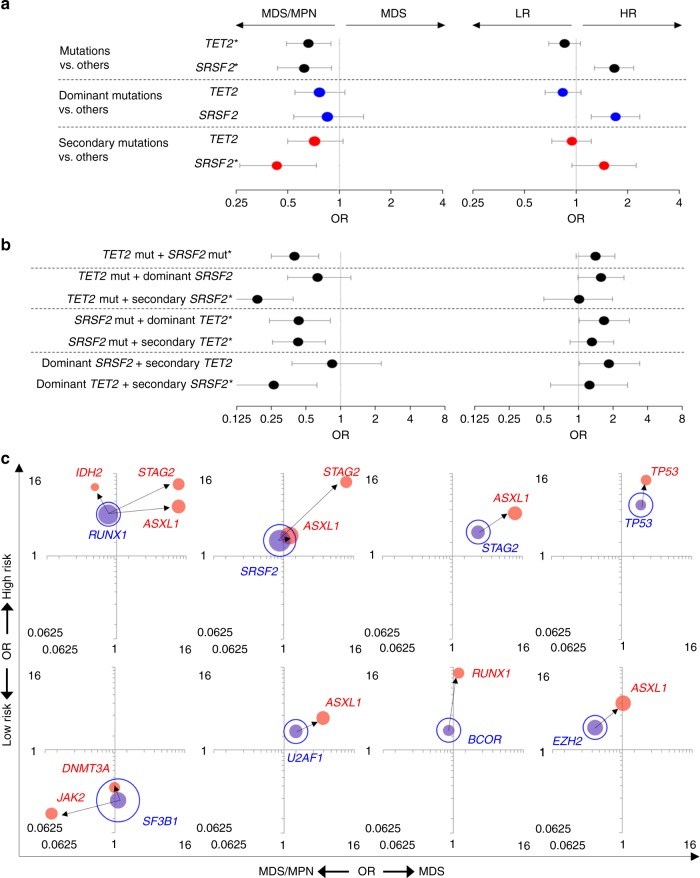
Clinical effects for frequent pairs of dominant and secondary mutations. **a**, **b** Clinical features for patients with *TET2* and/or *SRSF2* mutations. Forest plot shows the odds ratios (OR) of MDS vs. MDS/MPN (left side) and high (HR) vs. low-risk (LR) subtypes (right side) for genetic events of (**a**) single mutation and (**b**) pairs of mutations, respectively. The significance was determined by Fisher’s exact test; *P < 0.05 (MDS vs. MDS/MPN). Circle and error bars indicate OR and 95% confidence interval. Blue and red dots depict dominant and secondary mutations. **c** Clinical feature dependence on mutation combinations. Phenotypic features compared are MDS (*n* = 1583) vs. MDS/MPN (*n* = 226) on the *x*-axis, and low- (*n* = 1043) vs. high-risk (*n* = 766) groups on the *y*-axis. The area of each bubble indicates the frequency of mutations identified in 1809 MDS patients. Bubbles colored blue and red showed dominant and secondary mutations, respectively. Arrows indicate mutation sequence/ordering. All pairs of dominant and secondary mutations with significant phenotypic effects are highlighted by filled bubbles connected by arrows.

We then evaluated the impact of different types of mutations and their combinations on clinical phenotypes, including dichotomous morphological (MDS vs. MDS/MPN) features, progressive (low- vs. high risk) subtypes and survival. Depending on the position in the clonal hierarchy, the impact of specific mutations could vary in several genes (Supplementary Fig. 12). For instance, focusing on *TET2* and/or *SRSF2*, these mutations were associated with MDS/MPN phenotypes and high-risk disease (likely CMML) (Fig. 3a). *TET2* and *SRSF2* mutations were enriched in CMML patients [44% (53/121) and 32% (39/121), respectively], and 20% (24/121) of the CMML patients had both *TET2/SRSF2* mutations, a significantly frequent compared with the rest of MDS/MPN patients [2% (2/91) of the MDSMPN-U/RARS-T patients, *P* < 0.00001]. With regard to the clonal architecture, patients with *SRSF2* mutations as secondary hits were associated with MDS/MPN [OR 0.43 (0.26–0.74), *P* = 0.001]. Given that patients with dominant or secondary *TET2* mutations were unlikely to have MDS/MPN, one could hypothesize that phenotypic penetrance of *SRSF2* mutations would likely be greater than that of *TET2* mutations. In order to verify this hypothesis, we analyzed patients with both *SRSF2* and *TET2* mutations in detail (Fig. 3b). When patients with *TET2* mutations acquired secondary *SRSF2* mutations, they were significantly more likely to develop MDS/MPN [OR 0.19 (0.09–0.39), *P* = 2.7 × 10^−6^]. Conversely, patients with *SRSF2* mutations who had a secondary *TET2* mutation had similar association with MDS/MPN (*SRSF2* followed by dominant *TET2*; OR 0.43 or vice versa *SRSF2* followed by secondary *TET2*; OR 0.43). In additional analysis of specific pairs of mutations, patients with dominant *TET2* followed by secondary *SRSF2* mutations were significantly more likely to develop MDS/MPN that those with dominant *SRSF2* followed by secondary *TET2* mutations [OR 0.26 (0.12–0.62), *P* = .0013 vs. OR 0.84 (0.38–2.24), *P* = 0.7].

Focusing on other pairs of dominant and secondary mutations, 12 pairings affected risk characteristics (Fig. 3c; Supplementary Table 3). For example, dominant *RUNX1* mutations followed by secondary *ASXL1*, *STAG2*, or *IDH2* mutations were more likely associated with high-risk subtypes. Whereas, dominant *SF3B1* mutations followed by secondary *DNMT3A* or *JAK2* mutations were more likely associated with low-risk subtypes. Thus, distinct phenotypic features are due to specific dominant/secondary combinations, and multiple combinations can result in overlapping phenotypes. Of note is that secondary mutations in the same gene modify the phenotypic features. For example, patients with bi-allelic *EZH2* alterations were more likely associated with high-risk subtypes and MDS/MPN features compared mono-allelic *EZH2* (Supplementary Fig. 13).

**Fig. 4:**
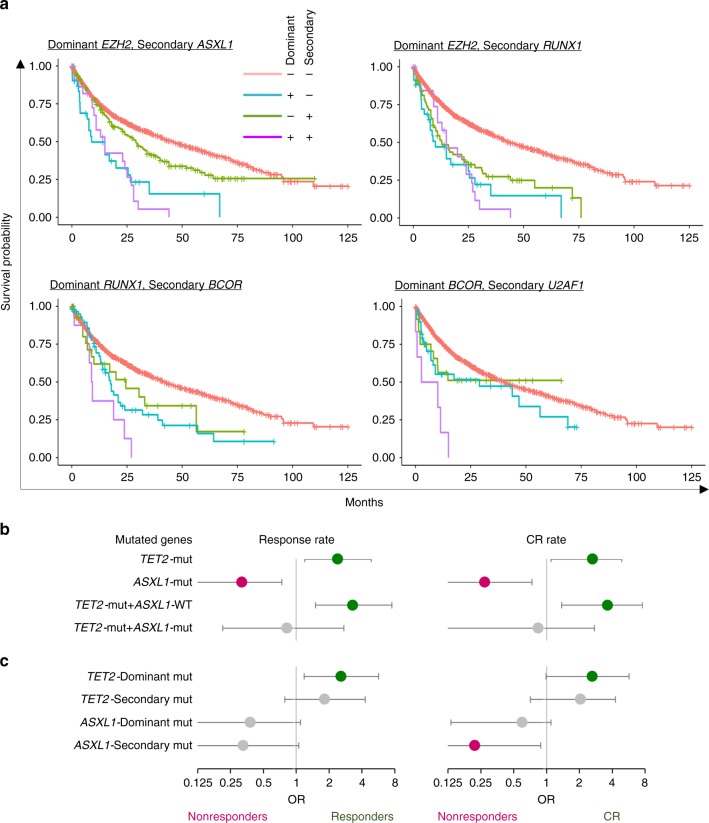
Survival and response of hypomethylating therapy for dominant and secondary mutations. **a** Kaplan–Meier curves for pairs of dominant and secondary mutations with significant effect. Representative four co-occurring pairs are shown in above the curves. Each graph depicts four different group results (both dominant and secondary mutation, only dominant mutations, only secondary mutations, and neither dominant nor secondary mutations). **b**, **c** Associations between the presence of mutations and responses for hypometylkationg therapy. Circle and whiskers indicate odds ratios (OR) and 95% confidence interval. Green circle depict responder (left panel) or complete response (CR) (right panel), whereas pink circle does nonresponders in both panels. Gray circle shows no significance. **b** Patients with *TET2* or *ASXL1* mutations. **c** Those with dominant or secondary mutations for *TET2* or *ASXL1* mutations.

Prior studies^30,31^ of MDS patients have demonstrated that the prognostic significance of *TP53* mutations depends in part on their VAF, with smaller clones having a less adverse impact. Of the 126 patients with *TP53* mutations in our cohort, 70 had a VAF > 0.4 and 56 had a VAF =< 0.4. The *TP53* mutant patients with a VAF > 0.4 had significantly shorter overall survival than those with a VAF =< 0.4 [median OS 8 vs. 17.8 months, hazard ratio (HR) 1.93 (95% CI: 1.23–3.04), *P* = 0.004] **(**Supplementary Fig. 14a**)**. In addition, *TP53* mutant patients with a VAF =< 0.4 had worse survival compared with *TP53* wild-type (WT) patients [median OS 17.8 vs. 44 months, HR 1.95 (1.36–2.79), *P* = 0.0003]. Focusing on clonal architecture, dominant and secondary *TP53* mutations were identified in 109 and 36 patients, respectively. Fifteen percent (19/126) of the *TP53* mutant patients had both a dominant and secondary mutation (Supplementary Fig. 14b). Patients with (i) both dominant and secondary, and (ii) only dominant *TP53* mutations had significantly shorter overall survival than those WT for *TP53* (median OS 7.33 vs 12.2 vs. 44 months, *P* < 0.0001). HR for both group vs. WT was 4.31 (2.53–7.33), whereas, those with dominant or secondary *TP53* mutations vs. WT were 2.92 (2.23–3.82) or 1.78 (0.98–3.23), respectively. In fact, 14 dominant/secondary mutations impacted survival (Supplementary Fig. 15). Focusing on 37 distinctive gene pairs, 9 co-mutations were associated with a poor prognosis (Fig. 4a; Supplementary Fig. 16). For instance, even though the frequent co-occurrence of a dominant *EZH2* mutation and secondary *ASXL1* or *RUNX1* mutations, they were associated independently with poor outcome. Whereas dominant *BCOR* and secondary *U2AF1* mutations did not affect survival, their co-occurrence was associated with shortened survival.

### Associations with response to hypomethylating therapy

Focusing on specific treatments, we identified 179 patients who were treated with hypomethylating agents (HMAs). Patients with either complete response (CR), partial response, or hematologic improvement were considered responders. According to IWG response criteria for MDS^32^, 37% (67/179) were responders, including 18% (33/179) achieving CR; 112 patients were nonresponders.

The presence of both *TET2* and/or *ASXL1* mutations was predictive of responsiveness (including CRs) or refractoriness to HMA therapy, respectively (Fig. 4b). For example, *TET2* mutations were associated with higher response rates than WT [53% (23/43) vs. 32% (44/136); OR 2.4 (1.2–4.9), *P* = 0.014], including CR, whereas, *ASXL1* mutations were associated with lower response rates. Prior studies^33^ showed that *TET2* mutant patients with WT for *ASXL1* had a higher response rate to HMA therapy, the results that were recapitulated in our cohort [61% (19/31) vs. 32% (48/148); OR 3.3 (1.5–7.5), *P* = 0.003]. On the other hand, these significant associations disappeared in patients with both *TET2* and *ASXL1* mutations (*P* = 0.76). This suggests that *ASXL1* mutations override the advantage of *TET2* mutations. In terms of clonal architecture, patients with dominant *TET2* mutations demonstrated higher response rates [56 vs. 33%; OR 2.6 (1.2–5.7), *P* = 0.017]; however, secondary *TET2* mutations did not (*P* = 0.16) (Fig. 4c). Although dominant or secondary *ASXL1* mutations could not predict response, patients with secondary *ASXL1* mutations were statistically unlikely to achieve CR [0 (0/14) vs. 25% (33/131), *P* = 0.04)]. *DNMT3A* and *TP53* mutations were not associated with response rates, while *U2AF1* mutations were associated with lower response rates (*P* = 0.047) (Supplementary Table 4).

### Distinct features of CH-derived and de novo MDS

Subclinical clonal expansions, referred to as clonal hematopoiesis (CH), are present in the blood of otherwise healthy individuals. While many CH-associated mutations occur in MDS, only a small proportion of asymptomatic individuals with CH progress to MDS. Because of the paucity of direct prospective data to project the fraction of CH-derived cases based on the spectrum of dominant hits, we stipulate that (i) a proportion of CH mutations will eventually serve as ancestral hits that manifest as MDS upon acquisition of additional genetic alterations, and (ii) MDS from antecedent CH may be an MDS disease subtype that is distinct from de novo MDS, characterized by more penetrant primary hits. Separating ancestral/dominant vs. secondary hits in MDS patients and comparing their frequencies to those (obtained through meta-analysis) in CH may enable molecular and clinical characterization of CH-related MDS.

**Fig. 5:**
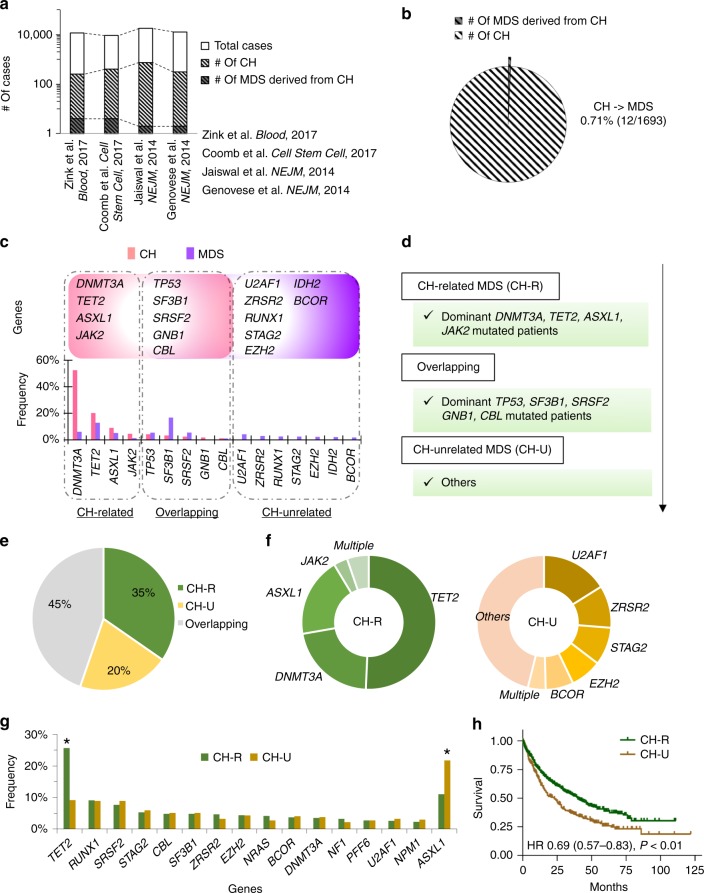
Characteristics of clonal hematopoiesis (CH)-related MDS. **a** The number of cases with sequenced, CH and MDS derived from CH in different four cohorts. Four papers are also shown. **b** Penetrance from CH to CH-derived MDS. Pie chart shows the number of cases with CH and CH-derived MDS in merged four cohorts. **c** Genes and frequencies of mutations in CH and MDS cohorts. Frequencies of mutation in the CH cohort and of dominant mutations in the MDS cohort are shown light pink and purple bar graphs, respectively; four genes (*DNMT3A, TET2, ASXL1*, and *JAK2*) which were mutated in MDS and frequently in CH are shown on the left. Five genes (*TP53, SF3B1, SRSF2, GNB1*, and *CBL*) which were mutated with similar frequency in both cohorts are shown in the middle. Seven genes that are frequently dominant mutants in MDS and uncommon in CH are shown on the right. These distinct groups are marked CH-related (CH-R), overlapping, and CH-unrelated (CH-U). **d** Estimating CH-related MDS. MDS patients with dominant mutations of *DNMT3A, TET2, ASXL1*, and *JAK2* were defined as CH-related. Those with *TP53, SF3B1, SRSF2, GNB1*, and *CBL* were defined overlapping. Finally, those with dominant mutations were defined as CH-unrelated. **e** Proportion of CH-R, CH-U and Overlapping MDSs. Pie chart shows MDS patients (*n* = 1809) divided into CH-R (*n* = 627), CH-U (*n* = 373), and overlapping MDS (*n* = 809). **f** Fractions of genetic mutations in CH-R and CH-U MDSs. Pie charts show fractions of genes with dominant mutations in CH-R (*n* = 627) and CH-U MDSs (*n* = 373). **g** Frequency of secondary mutations in CH-R (green) and CH-U MDS (yellow). *q *<* 0.01 (Fisher’s exact test *P*-values with Benjamini–Hochberg correction). **h** CH-R MDS cases have a better prognosis. Kaplan–Meier curves for overall survival among patients with CH-R MDS (green) and with CH-U MDS (yellow) are depicted. Tick marks indicate censored data.

For our analysis, the targeted panel covered 94.6% of CH mutations discovered by whole-exome sequencing^34^ (Supplementary Fig. 17a). We have also sequenced additional patients for the four genes (5.4%) which were omitted from our targeted panel genes, which resulted in only <0.9% of patients being affected (Supplementary Fig. 17b). When we compared mutated genes in a historical cohort of 1693 healthy CH individuals including 12 MDS patients who derived from CH to dominant mutations in our MDS patients (Fig. 5a, b; Supplementary Figs. 18, 19 and Supplementary Table 5), mutations in *DNMT3A, TET2, ASXL1*, and *JAK2* were more frequent in CH than in MDS (e.g., *DNMT3A*; 52 vs. 8%, *P* < 0.001; Fig. 5c; Supplementary Table 6). Hence, MDS patients with dominant mutations in these four genes were defined as CH-related MDS (CH-R; Fig. 5d). Other dominant mutations such as *U2AF1, RUNX1*, and *STAG2*, which were not identified in individuals with CH were deemed CH-unrelated MDS (CH-U). In between, mutations in *TP53, SF3B1*, *SRSF2*, *GNB1*, and *CBL* were identified with similar frequencies in both cohorts (e.g., *TP53*; 4 vs. 5%, *P* = 0.11), and such cases will be denoted as overlapping MDS patients, whereby founder mutations lead to directly to MDS or indirectly though CH.

We compared clinical, molecular, and demographic features of CH-R vs. CH-U. There were 627 (35%) CH-R and 373 (20%) CH-U cases, out of 1809 MDS patients (Fig. 5e); 97% of patients with CH-R had at least one dominant *TET2* (55%), *DNMT3A* (24%), or *ASXL1* (22%) mutation (Fig. 5f). The top five dominant mutations in patients with CH-U were *U2AF1* (19%), *ZRSR2* (12%), *STAG2* (10%), *EZH2* (9%), and *BCOR* (8%). In all, 54% of patients with CH-U had at least one of these mutations. Focusing on secondary mutations, patients with CH-R had a significantly higher frequency of secondary *TET2* mutations than those with CH-U (e.g., *TET2*, 26 vs. 9%, *P* < 0.001; Fig. 5g). In contrast, secondary *ASXL1* mutations were more frequent in CH-U [11 vs. 22%, *P* < 0.001]. CH-R cases were older and had more low-risk subtypes and normal karyotype than CH-U cases [e.g., average age 72 vs. 68-years old, *P* < 0.001; low-risk subtypes 58 vs. 44%, *P* < 0.001] (Table 3). Patients with CH-R also had a better prognosis than those with CH-U [HR 0.69 (0.57–0.83), *P* < 0.001; Fig. 5h].

**Table 3:** Clinical and molecular chracteristics of patients with CH-related (R) vs. -unrelated (U) MDSs.

		CH-R (*n* = 627)	CH-U (*n* = 373)	*P*-value
Age	Mean	72.4	67.5	<0.0001
	>60 y (%)	564 (92%)	283 (77%)	2.4 × 10^−10^
Sex	Male (%)	400 (63%)	254 (68%)	0.18
Diagnosis				2.0 × 10^−6^
	MDS	499 (80%)	264 (71%)	0.002
	MDS/MPN	86 (14%)	46 (12%)	0.56
	sAML	42 (7%)	63 (17%)	6.4 × 10^−7^
Risk classification				4.7 × 10^−5^
	Low risk	362 (58%)	165 (44%)	
	High risk	265 (42%)	208 (56%)	
Karyotypes				
	Normal	389 (62%)	186 (50%)	0.0002
	Complex	43 (7%)	33 (9%)	0.27
	−5/del(5q)	59 (9%)	38 (10%)	0.74
	−7/del(7q)	44 (7%)	41 (11%)	0.034
Number of mutations	Mean	3.2	2.6	<0.0001
Other mutations^a^				
	*RUNX1*	76 (12%)	60 (16%)	0.078
	*EZH2*	53 (8%)	45 (12%)	0.064
	*ZRSR2*	53 (8%)	55 (15%)	0.0022
	*STAG2*	47 (7%)	60 (16%)	3.2 × 10^−5^
	*U2AF1*	46 (7%)	81 (22%)	2.5 × 10^−10^
	*BCOR/L1*	39 (6%)	60 (16%)	1.0 × 10^−6^
	*IDH1/2*	37 (6%)	53 (14%)	1.0 × 10^−5^

^a^Frequently mutated genes, except for the nine genes identified in CHIP

## Discussion

Our goal was to study the clonal architecture of MDS to clarify the impact of mutations and their combinations on clinical phenotypes, and to assess the role of CH-associated mutations in frank MDS. For that purpose, we generated a well-annotated large cohort of untreated MDS patients that would allow us to account for the tremendous clinical heterogeneity and the corresponding genotypic diversity. Our initial results were concordant with previous studies with regard to the general mutational pattern^35^, validating the basis for subsequent analyses. In contrast to previous studies, we systematically explored correlations between mutational clonal hierarchy and clinical phenotypes.

After inferring clonal architecture by the stringent bioanalytic pipeline PyClone, which has been validated by single-cell sequencing, recurrently mutated genes could thus be characterized by the proportion with which they were dominant vs. secondary, i.e., initiating or supporting the clone; some genetic mutations are predominantly initiating, but with a propensity to occur at various stages of the clonal hierarchy. For instance, *TP53* or *SF3B1* mutations were more likely to be dominant, and *ASXL1 and KRAS* mutations were more like to be secondary. Dominant mutations defined distinct subgroups within which multiple secondary mutations accumulated. Among these pairings, we cataloged the most common combinations and characterized the extent to which they affected morphological and clinical phenotypes. In total, consequential 38 significant dominant/secondary pairs were identified. In addition to confirmatory relationships, such as dominant *SF3B1* and secondary *JAK2* mutations in RARS-T^36,37^, our analysis yielded several new associations.

The concept of dominant or founder lesions (*TET2*-, *SRSF2*-, or *TP53*-initiated disease etc.) would simplify molecular classification of MDS types. Specific dominant and secondary mutations, and their combinations significantly affected dysplasia (MDS vs. MDS/MPN), progression (higher- vs. lower-risk subtypes), survival and response of HMA therapy. For instance, *TET2* mutations were neutral with regard to the morphology, but they invited secondary hits such as *SRSF2*, resulting in subsequent drift toward an MDS/MPN phenotype and high-risk diseases. Similarly, dominant *EZH2* and secondary *ASXL1* or *RUNX1* mutations were associated with high-risk subtypes and cooperatively led to a poor prognosis. Moreover, analysis of the rank of *TP53* mutation within clonal structure is instructive: i.e., patients with dominant mutations, which reflects high clonal burdens, had worse prognosis compared with those with secondary mutations; those with both had the worst prognosis. This is consistent with bi-allelic *TP53* hits conveying typically the highest risk, as previously reported^30,31^. Finally, distinction of dominant hits can be also predictive of therapy responses, e.g., dominant *TET2* or secondary *ASXL1* mutations were more associated with response rate to HMA therapy than secondary *TET2* or dominant *ASXL1* mutations. Thus, dividing genetic mutations into dominant vs. secondary types may help us understand MDS pathogenesis and increase their predictive value as biomarker. Our results provide a catalog of dominant and secondary mutations and the phenotypic drives they exert and also how the impact of secondary hits depends on the ancestral hits preceding them. Irrespective of the dominant mutations, most secondary mutations increased the odds of advanced disease.

Obviously, resolution of identifying dominant and secondary hits solely by sequencing is a limitation of our study. Moreover, assessment of mutations at a single time point are not capable of definitive determination whether secondary mutations are present within the same subclone. Recent technologies enable single-cell analysis^2,38^ and genotyping of hematopoietic progenitor colonies of hematopoietic stem cells in MDS^27^. These methods are ideal for confirming clonal substructure, and tracking the clonal evolution of MDS^39^. While these technologies analytically are superior in individual patients^40^, given the time and cost investment, the PyClone approach is more practical clinically, and allows more generalizable results in large cohorts representative of molecular and clinical diversity of MDS.

Sequencing bone marrow DNA in patients with CH who later developed AML demonstrated that the leukemia was clonally derived from the previously identified CH^34^. Hence, acquired genetic mutations at the CH phase could represent early hits in MDS. In lieu of the very few prospective cases studied serially from CH to the evolution of MDS in the literature, we stipulated that mutations with a frequency lower in MDS than in CH point toward CH derivation, while those with present only in MDS (and absent in CH) evolve without prodromal CH phase. Accordingly, based on dominant hits in CH^18,19^ and MDS, we determined which patients should be classified as CH-R MDS or de novo MDS (CH-U MDS) and evaluated molecular and clinical differences between these two categories. Several reports of CH mutations shared with MDS yielded approximations of the fraction of mutations that were CH-derived. The majority of CH-related MDS had at least one dominant mutation of *DNMT3A, TET2*, and *ASXL1*, frequent secondary *TET2* and *ZRSR2* hits, older age, and better prognosis. Consistently, *DNMT3A, TET2*, and *ASXL1* mutations, which are most commonly associated with age-related clonal hematopoiesis, during complete remission phase did not increase risk of relapse in AML^41^, whereas other mutations such as *U2AF1* and *RUNX1* (absent in CH) could associate with relapse and lower rates of relapse-free survival^6,42^. These and other CH-unrelated ancestral hits impart faster growth/progression to symptomatic MDS, and this may have precluded their appearance in healthy elderly^43^. In contrast, CH mutations do not appear to be drivers and gain this property with time during aging or upon acquisition of secondary hits. In terms of epidemiologic considerations, CH-R MDS patients in longitudinal studies^18,22,44^ showed mutational profiles that are distinct from CH-U MDS.

Our targeted panel is lacking a few known recurrent genes (e.g., *PPM1D, MYD88, ATM*, and *STAT3*) with CH mutations, compared with the WES^34^. Therefore, a proportion of CH-U MDS cases might have preceded CH. To address this question, we searched for these mutations in an additional cohort, isolating them in <0.9% of patients. It suggests that the initial omission of these genes might not result in major misclassification. Indeed, *PPM1D*, while frequent in CH, was reported to be exceedingly rare in primary MDS and mostly present in therapy-related MDS/AML^45,46^. Consistent with these findings, the two patients with *PPM1D* mutations in our additional cohort had a previous history of chemotherapy or radiation therapy. Given that 1809 patients in our cohort had no history of chemotherapy or radiation, we anticipate *PPM1D* mutations to be extremely rare.

Another limitation of our CH vs. MDS comparison is that no confirmatory samples were analyzed before the time point of MDS diagnosis. Prospective analyses that include sequencing of serial samples from identification of CH to development of MDS in many patients would be optimal for revealing the landscape of cases with CH-derived MDS in greater detail. However, it might be difficult because of requirement large number of healthy donors and a long following-up time. We believe our hypothesis regarding the clinical and molecular characteristics of CH-derived MDS represent an alternative method useful in various clinical applications.

In conclusion, a stringent bioanalytic pipeline called PyClone estimated the clonal architecture in MDS patients and these results can be used to assign each patient to dominant events, and thus to subgroups sharing the event. Our results show that in the process of clonal evolution, various initial hits are preferentially followed by a specific spectrum of secondary hits that shapes phenotypic and biologic features of MDS. Focusing on ancestral/dominant events is instructive, it allows discrimination between MDS with antecedent CH and de novo MDS. Ancestral hits are the origins of MDS that determine unique characteristics in CH-R vs. CH-U MDS. Given the clinical characteristics of CH-R MDS, detecting and monitoring CH may be warranted, particularly as biomarkers better predict risks and as preventive methods are developed and become more readily available.

## Methods

### Patients

A total of 1809 patients with MDS (*n* = 1446), MDS/MPN (*n* = 212), and secondary AML (sAML) from MDS or MDS/MPN (*n* = 151) were screened and enrolled in this study (Table 1). Therapy-related MDSs were not included. In this paper 1169 patients (52%) were previously reported in the paper by Makishima et al., which included 2250 patients (Supplementary Fig. 1). Additional data such as copy-number alterations and read counts of mutations were needed for inferring accurate clonal structures by PyClone, therefore only half of the patients could be used. To increase statistical power, we added another 640 patients which were uniformly annotated to contain all elements required. In addition, patients were selected who had fully annotated outcomes with follow up and had pathomorphological evaluation available. All samples were obtained after written informed consent, according to protocols approved by the Institutional Review Board of participating institutions (Cleveland Clinic’s Institutional Review Board (IRB) and IRB-5024). In total, 212 patients were diagnosed with MDS/MPN; 121 patients (57%) with CMML, 47 patients (22%) with MDS/MPN-U, and 43 patients (21%) with RARS-T. All sAML cases were determined derived from MDS (*n* = 137) or MDS/MPM (*n* = 14) by WHO classification to dichotomize morphologic features^4^. All cases are separated into two groups based on IPSS-R scores (low risk as <=3.5 and high risk as >3.5) according to the transformation risk^47^, 58% of the patients (1043/1809) were low risk, whereas, 42% (766/1809) were high risk (Table 1). GL DNA was obtained from either buccal mucosa or CD3-positive T cells, which were purified from peripheral blood with or without prior culture in the presence of PHA and IL-2^8^. Tumor DNA was extracted from bone marrow or peripheral blood.

### Whole-exome sequencing

WES was performed as previously described^8,29^. Paired disease and normal GL DNA were used. Whole-exome capture was accomplished through hybridization of sonicated genomic DNA to a bait cDNA library synthesized on magnetic beads (SureSelect Human All Exon 50 Mb or V4 kit, Agilent Technologies). Captured targets were subjected to massively parallel sequencing using a HiSeq 2000 (Illumina) according to the standard protocol for 100-bp paired-end reads. Briefly, sequencing reads were aligned to the human genome (hg19) by a Burrows-Wheeler aligner (http://bio-bwa.sourceforge.net/). We used a GATK pipeline to extract candidate variants/polymorphisms and to remove sequencing errors. Validations were performed by Sanger or PCR amplicon sequencing as previously described^29^.

### Targeted sequencing

Targeted sequencing was performed using a TruSeq Custom Amplicon (Illumina) or a custom cRNA bait library (SureSelect; Agilent Technology) as previously described^29,37,48^. The common 36 genes were overlapped in both targeted regions (Supplementary Table 1). Sequencing libraries were generated according to an Illumina paired-end library protocol. The enriched targets were subjected to massive sequencing using Hiseq 2000 or Miseq sequencer (Illumina), with sufficient read coverage (Supplementary Fig. 2). Variants were annotated using Annovar^49^ and filtered by removing: (i) synonymous single-nucleotide variants; (ii) variants only present in unidirectional reads; (iii) variants in repetitive genomic regions (Supplementary Fig. 3). Only variants with minimum depth of 20 and with 5 positive, high-quality reads, were considered. A bioanalytic pipeline, devised in-house, as previously described^48^, was applied to identify somatic mutations by comparison with sequenced controls and mutational databases, such as dbSNP138^50^, 1000 Genomes^51^ or ESP 6500 database, and Exome Aggregation Consortium (ExAC)^52^. Mapping errors were removed by visual inspection with the Integral Genomics Viewer. Validation by Sanger sequencing or PCR amplicon sequencing was performed as previously described^48^. Variant allelic frequencies were adjusted according to zygosity and copy number, based on conventional metaphase karyotyping/single-nucleotide polymorphism array results^48^. An overall accuracy of our platform for detection of somatic mutations was estimated to be 98.7% (74/75) (Supplementary Fig. 4).

### Copy-number analysis

We employed three different platforms (Karyotyping, microarray, and digital copy-number analysis) for chromosomal alterations (Supplementary Fig. 5). In all, 95.7% (1782/1809) patients were evaluated copy-number alterations, of which 1014 patients were studied by all three platforms. One is the conventional metaphase karyotyping, chromosome preparations were G-banded using trypsin Giemsa, and karyotypes were described in 1759 patients according to the International System for Human Cytogenetic Nomenclature^53^. Second is single-nucleotide polymorphism (SNP) array karyotyping for confirming metaphase cytogenetics and detecting copy-number normal loss of heterozygosity was performed as previously described^10,54^. Briefly, Affymetrix 250K and 6.0 SNP arrays were used to evaluate copy number and loss of heterozygosity. Using our internal and a publicly available database (http://dgv.tcag.ca/dgv/app/home), the screening algorithm validated each lesion as somatic. Non-somatic lesions were excluded from further analysis. Affected genomic positions in each lesion were visualized and extracted using CNAG (v3.0) or Genotyping Console (Affymetrix)^55,56^. Third is digital copy-number analysis. Copy numbers for target exons were calculated, Let $d_j^{i,s}$ be the sequencing depth at the *i*th nucleotide of the *j*th exon in sample *s*, and the standardized depth of the *j*th exon is calculated as $D_j^s = k_s\mathop {\sum }\nolimits_i d_i^{j,s}$, where *k*_*s*_ is determined so as to satisfy $k_0 = \mathop {\sum }\nolimits_j D_j^s$ for a fixed constant *k*_0_ (= 1, for example). Correlation co-efficient (*R* = *R*^*s,t*^ between two vectors $D_i^s\,and\,D_i^t$ was calculated between sample *s* and each of the 443 samples with completely normal copy numbers in aCGH (*t* *=* 1, 2, 3, …, 443), respectively, through which the best *m*_0_ correlations were identified between samples *T*_*m*_ (*m* = 1, 2, 3, …, *m*_0_). The copy numbers of the *i*th target exon of sample s ($Cn_i^s$) was calculated as $Cn_i^s = D_i^s/\hat D_i^s$, where $\hat D_i^s$ was provided by averaging *m*_0_ samples, $\hat D_i^s = \mathop {\sum }\nolimits_{m = 1}^{m_0} D_i^{t_m}$/*m*_0_ with *m*_0_ = 12. Copy numbers were calculated for those exons, showing mean depth > 500. Circular binary segmentation was used to identify discrete copy-number segments, using DNACopy by which segmented copy number ($\widehat {Cn}_i^s$) was defined for the *i*th exon of sample *s*. Distribution of $\widehat {Cn}_i^s$ was calculated for all samples, and those exons showing $\left| {\widehat {Cn}_i^s - E\left( {\widehat {Cn}_i^s} \right)} \right| \, > \, 4SD$ were thought to have copy-number losses or gains, respectively.

### Estimation of tumor cell fraction

The estimated tumor cell fraction harboring the relevant mutation (TCFmut) was calculated from total copy number (TCN) of the region, minor allele-specific copy number (AsCN), and observed variant allele frequency (VAFobs) as follows; TCFmut = TCN × VAFobs (for deletions), TCFmut = 2VAFobs-1 + AsCN [for UPD when VAF > 0.5(1-AsCN) (UPD occurred later)], TCFmut = 2VAFobs [for UPD when VAF < 0.5(1-AsCN) (mutation occurred later)] TCFmut = 2VAFobs (for regions without copy-number changes or UPD). For gains, since we cannot accurately estimate the correct TCN and tumor cell fractions, we tentatively assumed TCN as three, and accordingly tumor cell fractions can be estimated as TCFmut = TCN × VAFobs.

### Clonal structure inference

To detect subpopulations, clustering analysis of mutations was performed according to the Beta Binomial emission model implemented in PyClone^28,57^. Mutations in repetitive regions or indels, for which VAFs were poorly estimated, were not used for the analysis. Clustering was performed using a dynamic Tree Cut procedure. Density was used to model read counts (pyclone_beta_binomial), and PyClone ver 0.13.0 was run with 10,000 iterations and a burn- in of 1000 with default setting^29,58^. A configuration file was generated for each patient by using the total copy-number option. The remaining parameters were set as follows: base measure parameters: alpha = 1, beta = 1; concentration value = 1; prior shape = 1.0, rate = 0.001; for each sample, the error rate parameter was set to 0.001. Clonal hierarchy was reduced to a one-dimensional ordered 2-level feature space of primary/dominant, and secondary mutations (Fig. 1d). Mutations belonging to the largest clone (Pyclone cluster 1) were defined as dominant mutations and other clones as secondary mutations (Fig. 1e; Supplementary Fig. 7 and Supplementary Data 2).

### Correlations of mutations

Frequent mutations and copy-number alterations (>2%, *n* = 30) were assessed for mutual correlation. Any combination of these variants was exhaustively tested in a pairwise manner using Fisher’s exact test, and multiple testing was corrected with the Benjamini–Hochberg *q*-value (assumed significant when *q*-value < 0.01 for coexistence and <0.25 for exclusion). Significant correlations were plotted with transition colors (magenta for positive and green for negative correlations), together with circle diameters indicating the degree of significance.

### Association morphologic and features with mutations

The 1809 patients were dichotomized by morphologic features (MDS (*n* = 1583) vs. MDS/MPN (*n* = 226)) and by progression to AML (low risk (*n* = 1043) vs. High risk (*n* = 766)) by the criteria as shown above in patients. The bubble plot shows both odds ratios on the *x*- and *y*-axes, respectively. Odds ratios for specific combinations of dominant and secondary mutations were calculated using the following steps; (i) count numbers of patients with (A) both dominant and secondary mutations, and (B) neither dominant nor secondary mutations; (ii) 2 × 2 (A) vs. (B) tables were dichotomized by morphology and progression to AML; (iii) odds ratios were then estimated. Combinations with significance in either morphology or progression to AML are shown (Fig. 3b, c; Supplementary Table 3).

### CH-related MDS detection

Meta-analysis of mutation distribution within various large CH cohorts^19,34,59–61^ showed that the top eight most mutated genes account for nearly all of CH (Supplementary Figs. 17, 18). We then hypothesized that if a fraction of MDS originates from CH, the compositions of dominant mutation should be similar. *DNMT3A, TET2, ASXL1,* and *JAK2* mutations were significantly higher in CH compared with our MDS cohorts, hence MDS patients with dominant mutations of these four genes were defined as CH-related MDS (Fig. 5c, d). Mutations of the remaining five genes (*SF3B1, SRSF2, TP53, GNB1,* and *CBL*) were identified in both cohorts, so MDS patients with them were Overlapping, whereas those with other dominant mutations such as *U2AF1, RUNX1*, and *STAG2* were CH-unrelated MDS. Those without any mutations were overlapping because they could not be separated by these criteria.

### Statistical analysis

Comparisons of proportions were performed by using two-sided Fisher’s exact tests. Paired data were analyzed by the Wilcoxon signed-rank test. Continuous variables were compared using the Mann–Whitney *U* test. Kaplan–Meier methods were used to plot survival. The log-rank test was used to compare survival curves. Univariate and multivariate Cox proportional hazards survival analyses were also conducted. Analyses were performed with R (https://www.r-project.org), SPSS software (IBM), and Prism (GraphPad). Significance was determined at a two-sided α level of 0.05, except for *P*-values in multiple comparisons, which were adjusted according to the method described by Benjamini and Hochberg^62^.

### Reporting summary

Further information on research design is available in the Nature Research Reporting Summary linked to this article.

## Supplementary information

Supplementary Information

Description of Additional Supplementary Files

Supplementary Data 1

Supplementary Data 2

Reporting Summary

## Data Availability

Genome data that support the findings of this study have been deposited through the database of Genotypes and Phenotypes (dbGaP) in the NCBI under accession number phs001898.v1.p1. All other remaining data are available within the Article and Supplementary Files, or available from the authors upon request.
