# Supplementary material for: Invariant patterns of clonal succession determine specific clinical features of myelodysplastic syndromes
Source: Nat Commun. 2019 Nov 26;10:5386. doi: 10.1038/s41467-019-13001-y (PMC6879617; doi:10.1038/s41467-019-13001-y)
Supplement: Supplementary file 1 — Supplementary Information [file 41467_2019_13001_MOESM1_ESM.pdf]

Supplementary Information

Invariant patterns of clonal succession determine specific clinical features of myelodysplastic syndromes

Nagata et al.

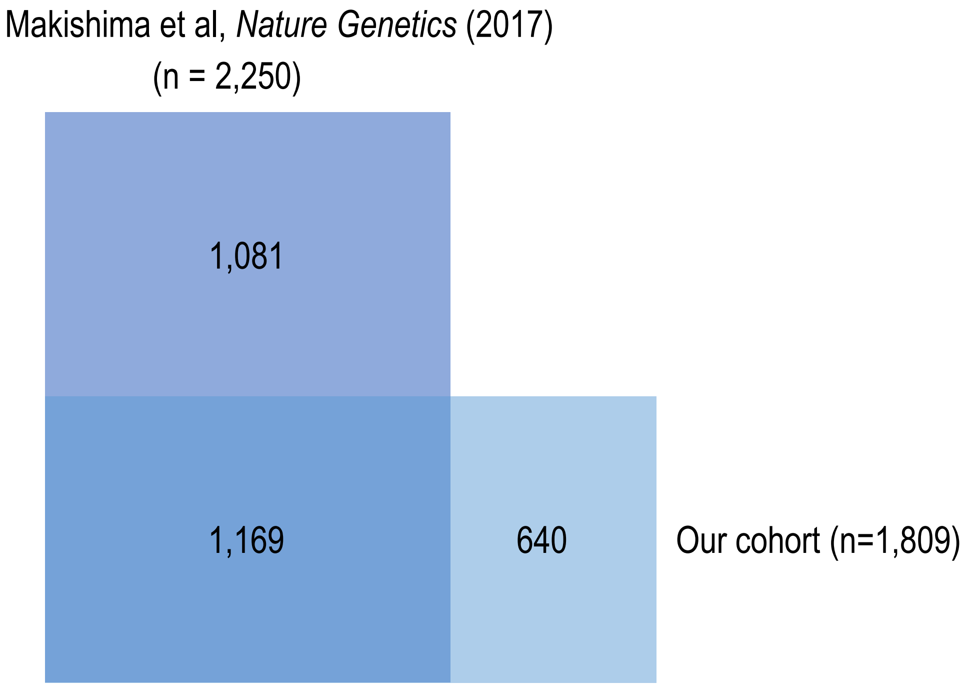

Supplemental Figure 1. The fraction of newly analyzed samples in this study

There were 640 (35%) new samples analyzed in addition to 1,169 (65%) which were a part of the previous papers [[1]Makishima et al, *Nature Genetics* (2017)]. 52% of patients (1,169/2,250) were overlapping between the two studies. Patients were selected who had fully annotated sequencing data, outcomes with follow up, and pathomorphological evaluation available were selected for this study.

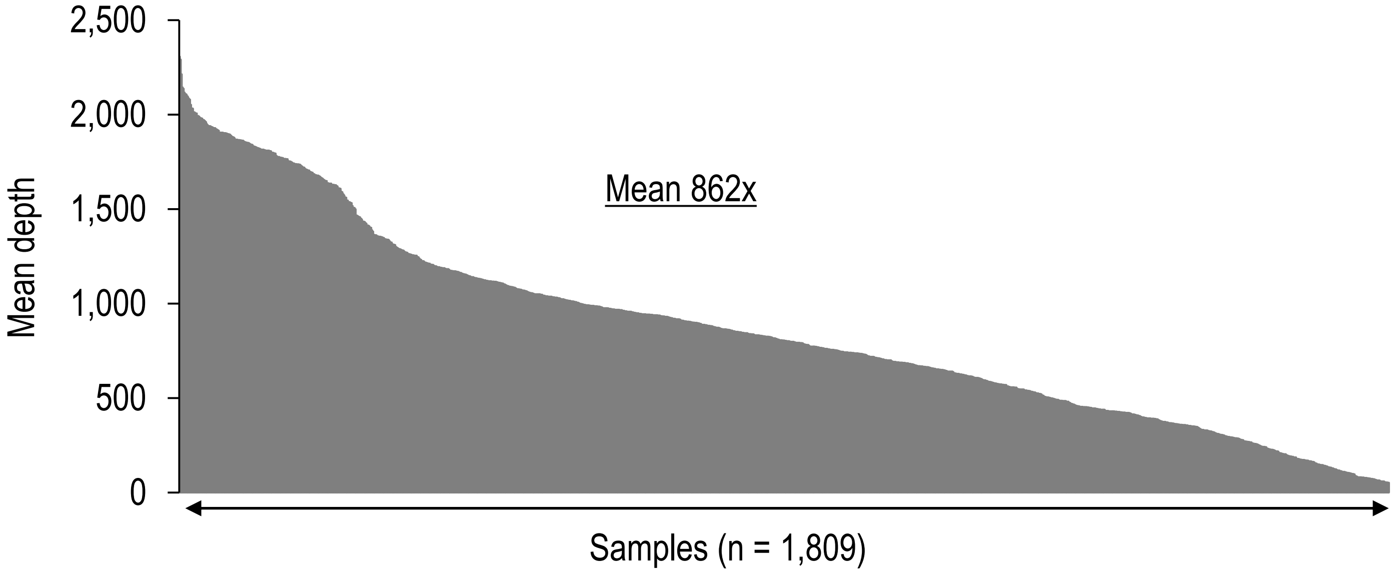

Supplemental Figure 2. Sequencing depths

Bar graphs depict mean read counts (depth) of targeted regions in each sample.

1.Align fastq files to the human genome reference (genome assembly GRCh37 (hg19)

2. Remove PCR duplicates by MarkDuplicates in Picard (Broad Institute)

3. Keep only reads and bases with high quality (Read quality>25, Base quality >30)

4. Variants with $\geq$5 mutated read counts and $\geq$20 total read counts

5. Remove below variants;
i) synonymous and ambiguous single nucleotide variants;
ii) variants only present in unidirectional reads;
iii) variants in repetitive genomic regions.
iv) variants found in dbSNP138, 1000 Genomes or ESP 6500 database, and ExAC.
v) Variants with mapping error confirmed by visual inspection with the IGV

6. Confirmation of recurrent mutations at canonical nucleotide position which were registered COSMIC and rescue them

7. 299 out of the 300 randomly selected mutations were successfully validated (99.7%) on an independent sequencing platform, i.e. using a MiSeq instrument (Illumina).

8. 3,971 mutations in 36 genes were finally identified in 1,809 MDS patients.

Supplemental Figure 3. A bio-analytic pipeline of mutation for targeted sequencing

Gray squares depict each step for making the mutation list in targeted sequencing.

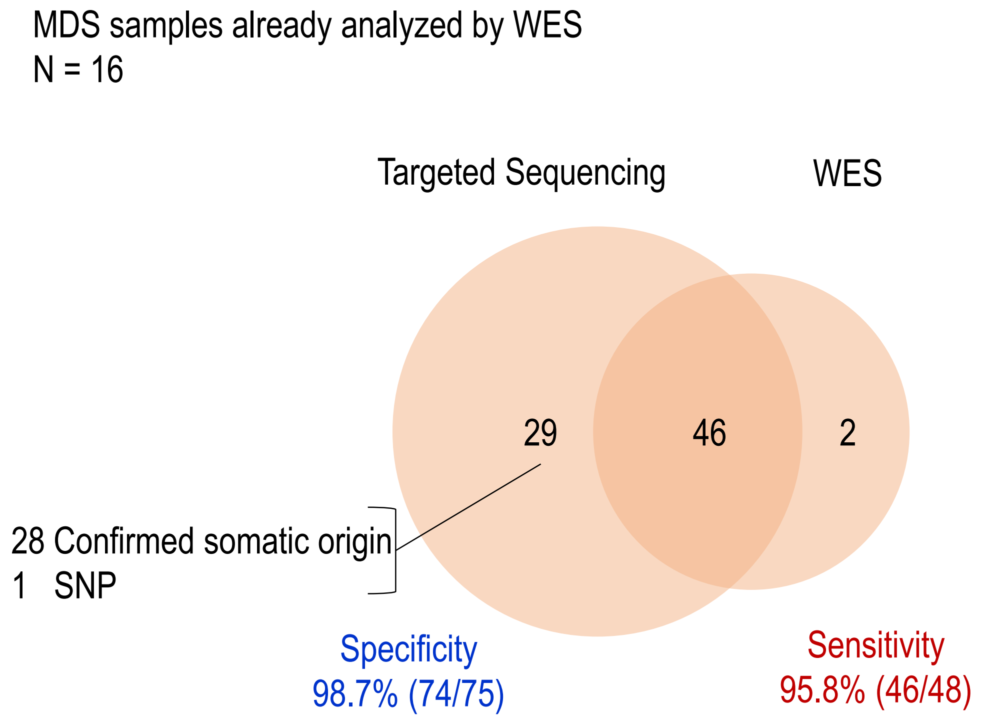

Supplemental figure 4. Validation for accuracy of somatic origins in targeted sequencing

To evaluate the accuracy of somatic mutations found by targeted tumor sequencing, we performed targeted sequencing of 16 tumor samples that had previously analyzed tumor/normal paired by whole exome sequencing (WES), including 12 cases were previously reported ([2]Yoshida et al., *Nature*. 2011; 478:64-9) were used for verification.

For the 16 MDS samples, our targeted sequencing approach and mutation calling algorithm successfully captured 46 out of the 48 previously confirmed somatic mutations by exome sequencing (sensitivity: 96%). The remaining 2 mutations escaped from mutation calling. This was because they harbored an allele frequency within the range to be excluded by our pipeline, i.e. variant frequencies between 0.45-0.55, and had not been registered in the COSMIC database or appeared in a set of normal DNA at >0.0025 of allele frequencies. On the other hand, this deep-sequencing approach also called an additional 29 SNVs in the 16 MDS samples, of which 28 variants were confirmed as somatic mutations using normal DNA as comparison (specificity 99%).

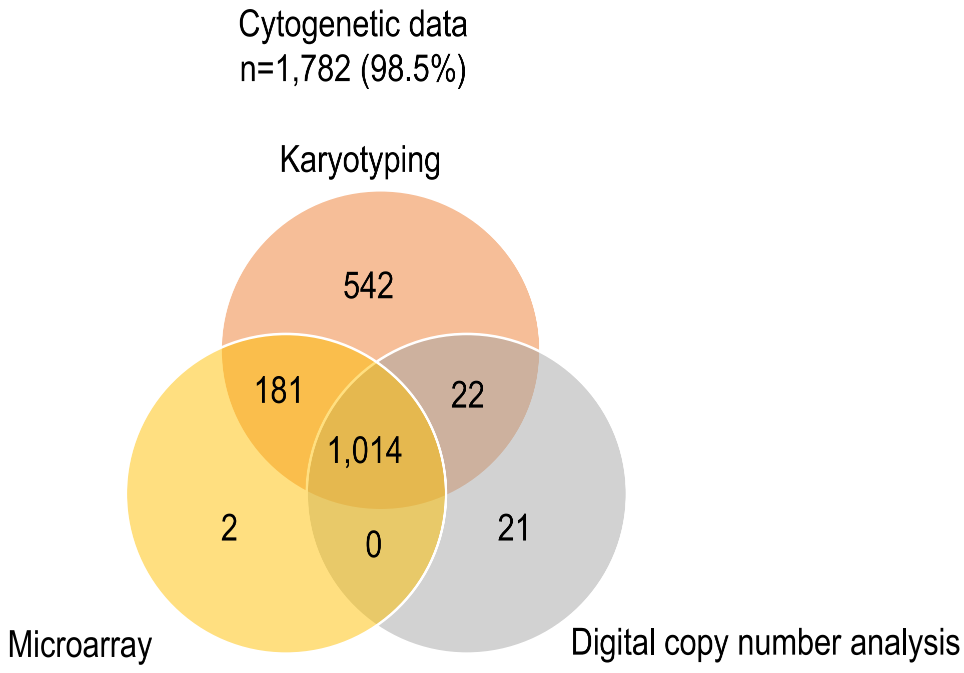

Supplemental Figure 5. Copy number alteration analysis

Karyotyping, Microarray, Digital copy number analysis were subjected in MDS patients

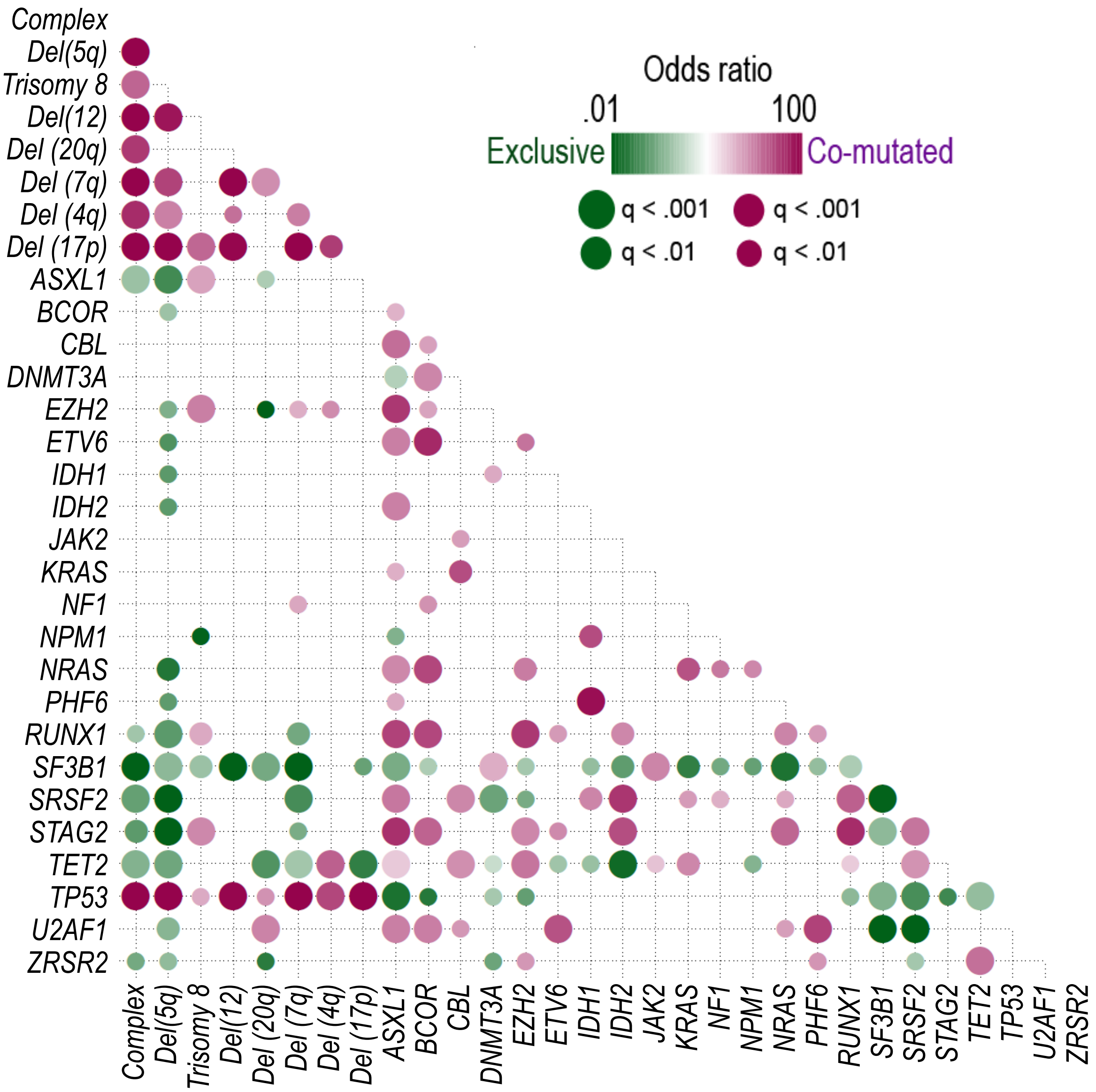

Supplemental Figure 6. Significant association in frequent mutations/copy number alterations

Recurrent mutations or copy number alterations (>2%, n=30) are given in the y- and x-axes, respectively. Co-occurrence and mutually exclusivity are encoded in purple and green color gradients, respectively. Circle sizes encode q-values (Fisher's exact test P values with Benjamini–Hochberg corrections).

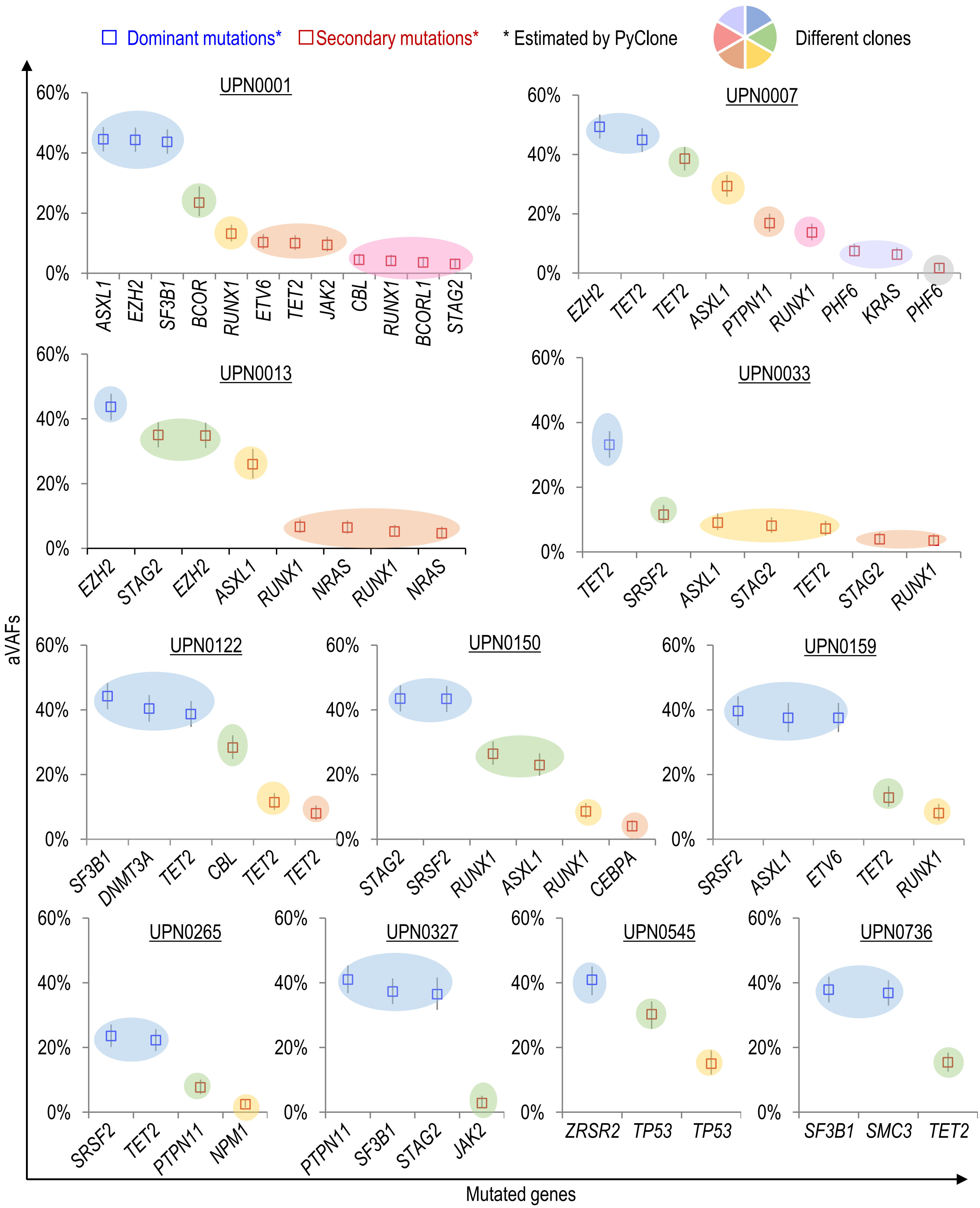

Supplemental Figure 7. Representative samples with multiple clones

Copy-number adjusted variant allele frequencies (aVAFs) (y-axis) and mutated genes (x-axis) in 11 illustrative samples were shown. Blue and red squares depict dominant and secondary mutations, respectively. Circles with different colors show the different clones estimated by PyClone.

Genes	Ranking of TS	Ranking of WES	TS (n = 1,584)	WES (n = 225)
SF3B1	1	2	22%	9%
TET2	2	1	20%	14%
SRSF2	3	4	9%	7%
DNMT3A	4	5	9%	5%
ASXL1	5	6	8%	5%
U2AF1	6	7	6%	4%
TP53	7	3	6%	8%
RUNX1	8	9	4%	3%
ZRSR2	9	10	4%	3%
STAG2	10	11	4%	2%
EZH2	11	8	3%	4%

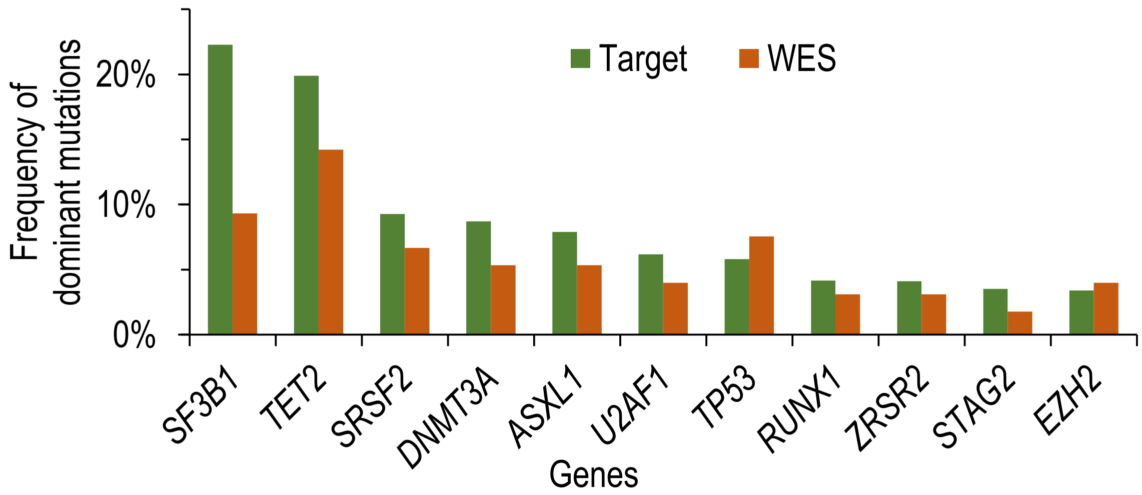

Supplemental Figure 8. Top 10 dominant mutations in whole exome (WES) and targeted sequencing (TS) cohorts

Frequent dominant mutations (top 10) are shown in both WES (n=225) and TS (n=1,584). 9 out of top 10 genes were overlapped between WES and TS cohorts.

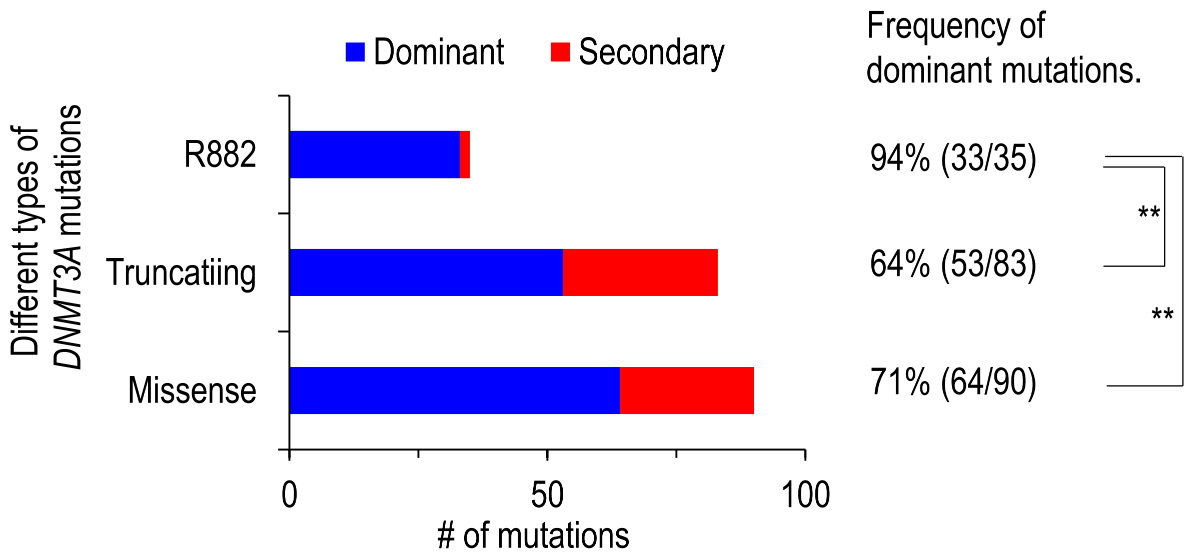

Supplemental Figure 9. Association different hotspot mutations and clonal hierarchy in *DNMT3A* mutations

DNMT3A mutations (n=208) are separated to three different location or types; Arg882 (n=35), missense mutations in other location (n=83), and truncating mutations (n=90). Horizontal bar graphs depicts fraction of dominant, and secondary mutations in each type. ** *P* < 0.01, Fisher's exact test.

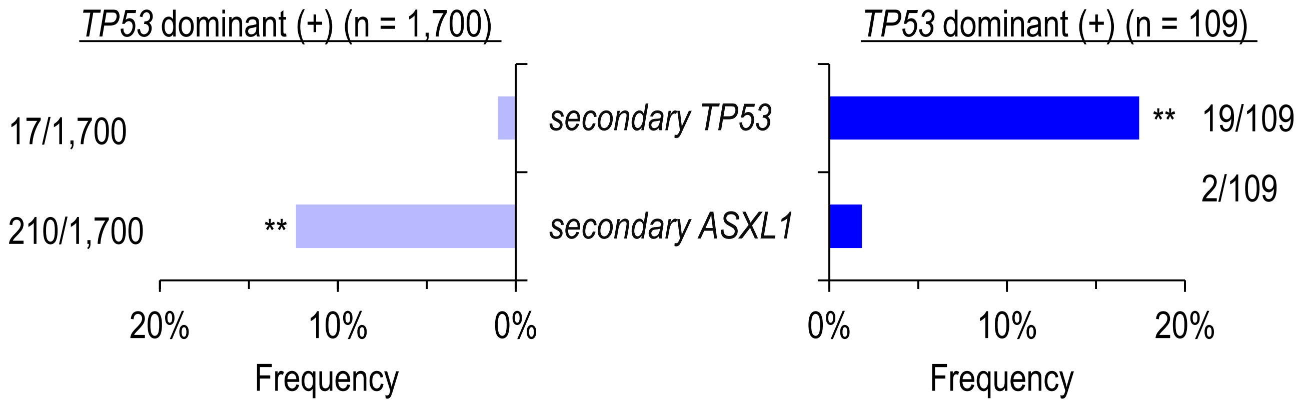

Supplemental Figure 10. Dominant *TP53* mutations determined secondary mutations

Comparison of secondary mutations between patients with dominant *TP53* mutations (n=109) vs. without them (n=1,700). Significant pairs are shown. **q < 0.01, Fisher's exact test *P* values with Benjamini–Hochberg correction.

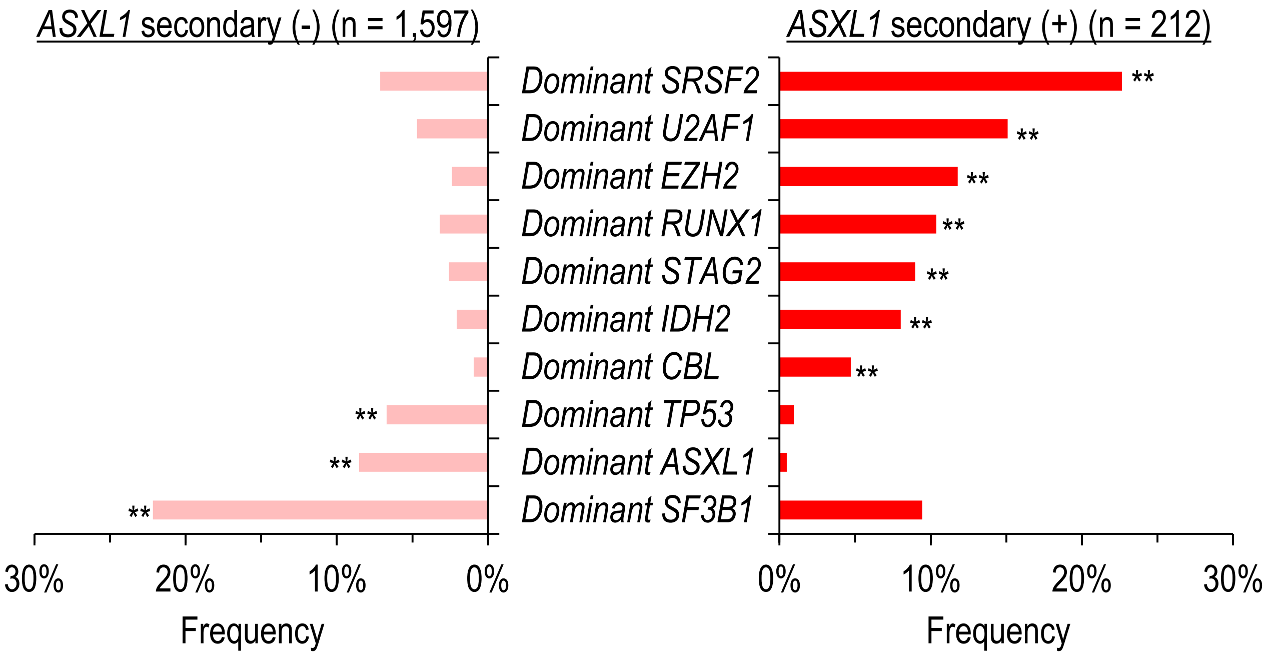

Supplemental Figure 11. Secondary *ASXL1* mutations emerged in particular dominant mutations

Comparison of dominant mutations between patients with secondary *ASXL1* mutations (n=166) vs. without them (n=1,643). Significant pairs are shown. **q < 0.01, Fisher's exact test *P* value with Benjamini–Hochberg correction.

MDS/MPN LR — MDS HR

Gene	Dominant (D) or Secondary (S) mutations	MDS *vs.* MDS/MPN	HR *vs.* LR
JAK2	S	0.11	0.53
JAK2	D	0.22	
CBL	D	0.30	
EZH2	D	0.34	2.13
KRAS	S	0.35	
NRAS	D	0.35	6.43
NRAS	S	0.40	3.31
SRSF2	S	0.43	
CBL	S	0.46	
TP53	D		5.61
TP53	S		4.20
RUNX1	D		4.09
IDH2	S		3.92
PTPN11	S		3.92
STAG2	S		3.20
PHF6	D		2.75
RUNX1	S		2.70
EZH2	S		2.56
IDH2	D		2.48
NPM1	D		2.45
U2AF1	S		2.30
STAG2	D		2.25
ASXL1	S		2.14
ETV6	S		2.07
NPM1	S		2.07
BCOR	D		1.94
BCOR	S		1.89
U2AF1	D		1.88
ASXL1	D		1.83
SRSF2	D		1.70
ZRSR2	S		0.43
SF3B1	S		0.32
SF3B1	D		0.18

Supplemental Figure 12. Disease phenotype between dominant *vs.* secondary mutations

Odds ratio between patients with mutations vs. without them are statistically analyzed in terms of dichotomized morphologies (MDS vs. MDS/MPN) or risk-subtypes (High risk vs. Low risk), respectively. Parameter with significance (q < 0.1, Fisher's exact test with Benjamini–Hochberg correction) are shown. Group1 (MDS or HR) and group2 (MDS/MPN or LR) are encoded in brown and green color gradients, respectively.

a

Types of *EZH2* alterations	MDS	MDS/MPN
Mono-allelelic alteration	183	22
Bi-allelic alterations	49	19

OR 0.31, 95%CI (0.16-0.63)

b

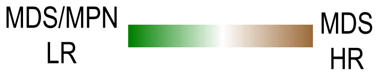

G1	G2	Genes	MDS vs. MDS/MPN	HR vs. LR
Bi-allele	Mono-allele	*EZH2*	0.31	
Bi-allele	Mono-allele	*TET2*		2.47
Truncating	Missense	*TET2*		2.29

Supplemental Figure 13. Disease phenotype between different configurations and types

(a) # of patients with bi-allelic or mono-allelic *EZH2* alterations are shown in different columns in terms of dichotomized morphologies (MDS vs. MDS/MPN), respectively. Odds ratio (OR) was calculated by Fisher's exact test. **(b)** OR between patients with group1(G1) event vs. group2(G2) are statistically analyzed in terms of dichotomized risk-subtypes (High risk vs. Low risk), respectively. Parameter with significance (q < 0.1, Fisher's exact test with Benjamini–Hochberg correction) are shown. Group1 (MDS or HR) and group2 (MDS/MPN or LR) are encoded in brown and green color gradients, respectively.

a

b

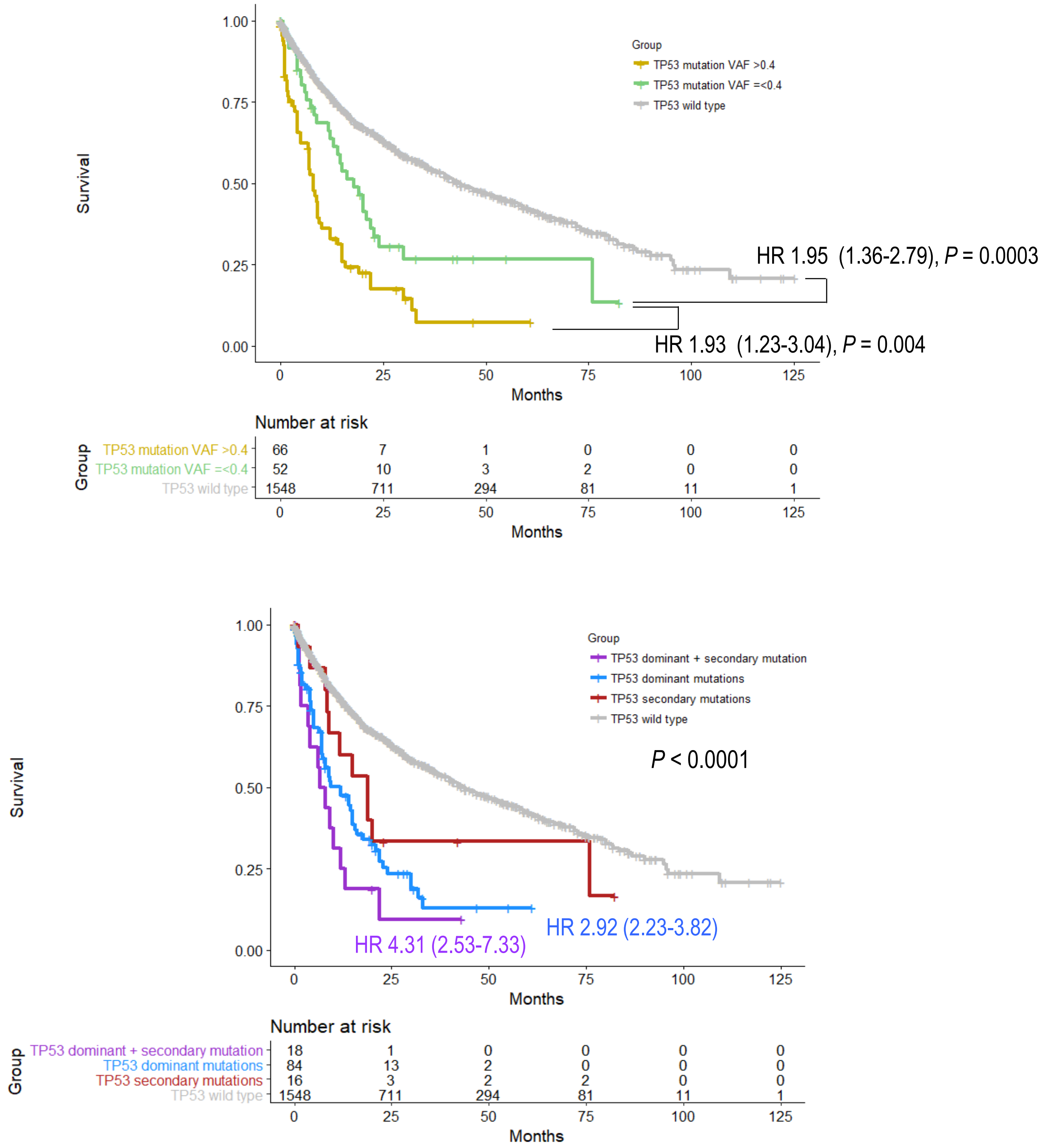

Supplemental figure 14. Overall survival, according to *TP53* mutational statues

(a) Effects of variant allele frequencies (VAFs) of *TP53* mutations. Kaplan-Meier curves for patients with *TP53* mutations VAF >0.4 (yellow), =< 0.4 (green) and wild type (gray) are depicted. The numbers of patients at risk are shown at indicated time points in the bottom. (b) Effects of combined *TP53* dominant and secondary mutations. Kaplan-Meier curves for clonal architecture of *TP53* mutations are presented.

Genes	Types of mutations	Hazard ration (HR) and 95% CI	*P*	# of mutated patients	*q*
TP53	Dominant	3.08 (2.41-3.93)	>1.0E -15	109	>1.0E -15
SF3B1	Dominant	0.46 (0.38-0.57)	7.86E-14	374	1.651E-12
RUNX1	Secondary	2.22 (1.74-2.84)	1.62E-10	109	2.268E-09
EZH2	Dominant	2.69 (1.97-3.68)	5.33E-10	63	5.597E-09
NRAS	Secondary	2.51 (1.73-3.65)	1.19E-06	47	9.996E-06
STAG2	Secondary	1.92 (1.45-2.53)	5.26E-06	84	3.682E-05
TP53	Secondary	2.49 (1.67-3.72)	7.23E-06	36	4.338E-05
RUNX1	Dominant	1.96 (1.45-2.65)	1.31E-05	73	6.878E-05
ASXL1	Dominant	1.59 (1.26-2.01)	8.41E-05	137	0.0003925
ASXL1	Secondary	1.45 (1.19-1.75)	0.000171	212	0.0007182
NRAS	Dominant	2.21 (1.40-3.48)	0.000655	28	0.0025009
PTPN11	Secondary	2.55 (1.47-4.42)	0.000836	23	0.002926
BCOR	Dominant	1.86 (1.28-2.72)	0.00122	48	0.0039415
U2AF1	Dominant	1.54 (1.18-2.00)	0.00143	107	0.00429

Supplemental Figure 15. Survival on patients with dominant or secondary mutations

Hazard ratio (survival) between patients with mutations vs. without them are statistically analyzed , parameter with significance ($q < 0.01$, Fisher's exact test with Benjamini–Hochberg correction) are shown.

a

Dominant	Secondary	P value	Hazard ratio and 95%CI	# of patients with dominant mutations	# of patients with secondary mutations	# of patients with both dominant and secondary mutations	Q value
TP53	*TP53*	3.26E-07	3.99 (2.35-6.78)	109	36	19	1.21E-05
BCOR	*U2AF1*	7.29E-06	6.33 (2.83-14.17)	48	32	6	1.35E-04
EZH2	*TET2*	9.02E-06	2.98 (1.84-4.82)	63	258	22	1.11E-04
EZH2	*ASXL1*	9.58E-06	2.74 (1.75-4.27)	63	212	25	8.86E-05
EZH2	*RUNX1*	3.86E-05	2.68 (1.67-4.28)	63	109	23	2.86E-04
RUNX1	*BCOR*	0.000437	3.5 (1.74-7.03)	73	45	8	0.002
RUNX1	*ASXL1*	0.000649	2.31 (1.43-3.74)	73	212	22	0.003
EZH2	*FLT3*	0.00153	6.29 (2.02-19.6)	63	18	5	0.006
EZH2	*STAG2*	0.00179	3.04 (1.51-6.11)	63	84	10	0.007

b

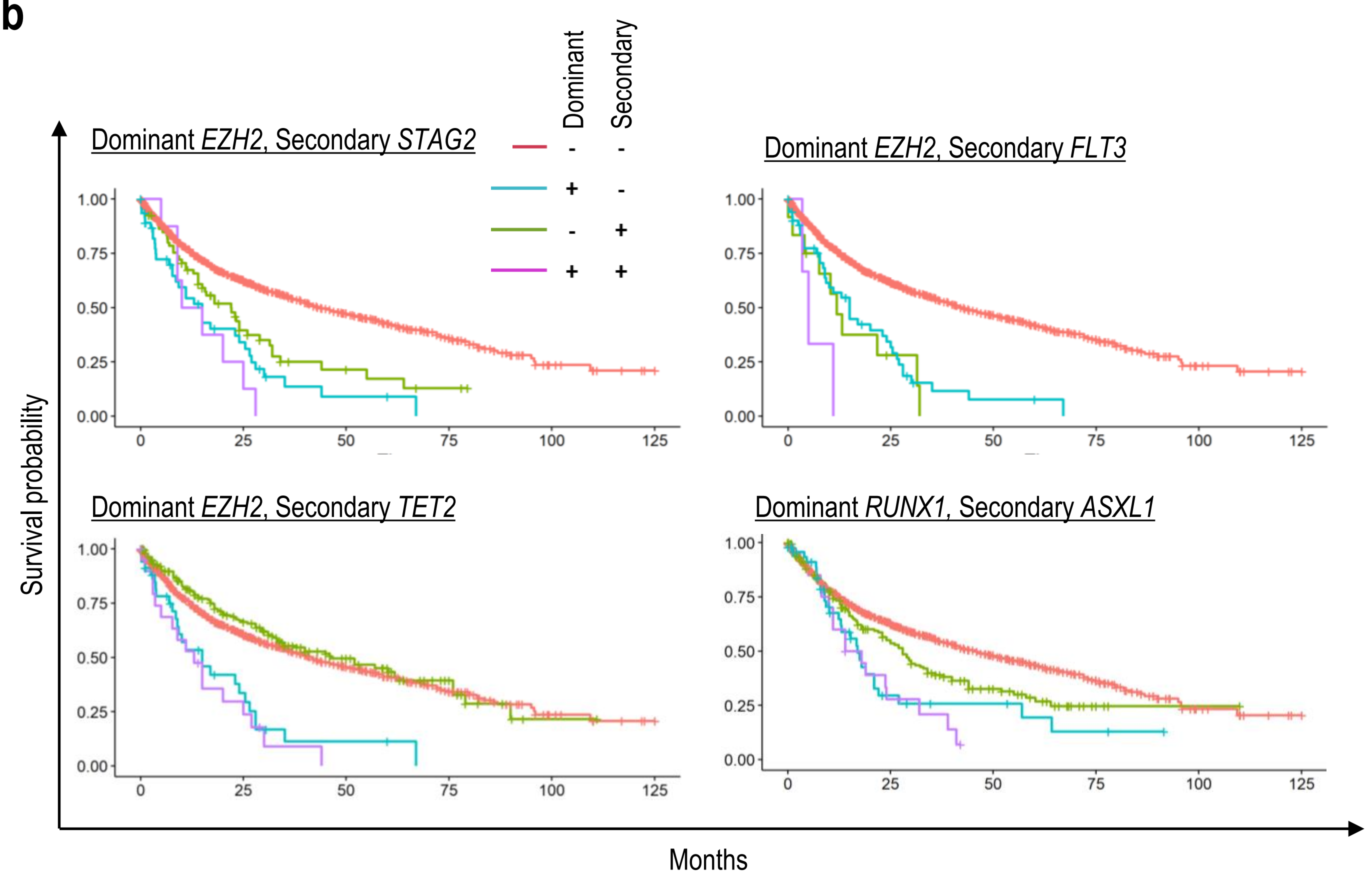

Supplemental Figure 16. Survival on patients with pairing of dominant/secondary mutations

(a) Hazard ratio (survival) between patients with paring of dominant and mutations vs. without dominant nor secondary mutations are statistically analyzed, 9 significant paring (q < 0.01, Fisher's exact test with Benjamini–Hochberg correction) are shown. (b) Kaplan-Meier curves for pairs of dominant and secondary mutations with significant effect

a

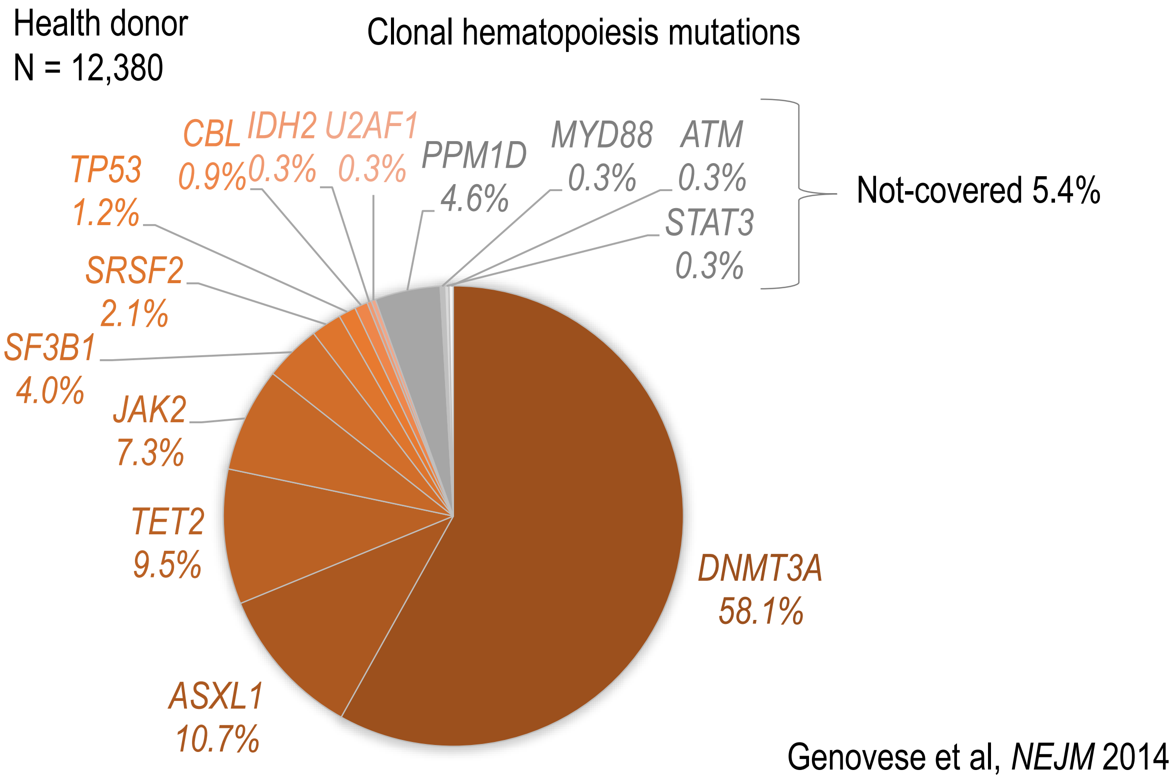

b

Genes	Frequency	Mutated/Sequenced
PPM1D	0.50%	2/397
MYD88	0.00%	0/297
ATM	0.89%	2/225
STAT3	0.65%	6/930

Gene	RefSeq	AA change	VAFs
PPM1D	NM_003620	p.V464Sfs*11	8.60%
PPM1D	NM_003620	p.P530fs	7.63%
ATM	NM_000051	p.K2440N	53.70%
ATM	NM_000051	p.L1327fs	20.36%
STAT3	NM_003150	p.Y640F	6.20%
STAT3	NM_003150	p.Y640F	37.30%
STAT3	NM_003150	p.K658delinsYK	5.2%
STAT3	NM_003150	p.K658delinsYK	16.8%
STAT3	NM_003150	p.Y640F	20.0%
STAT3	NM_003150	p.G618R	37.57%

Supplemental Figure 17. Comparing genetic mutations between clonal hematopoiesis (CH) and MDS cohorts

(a) 94.6% of CH mutations which reported in the paper ([3]Genovese et al, *NEJM* 2014) which used whole exome sequencing were covered in our targeted panels. 4 genes (5.4%) were omitted in our panel. (b) Frequency and mutational information for the 4 genes in additional cohort.

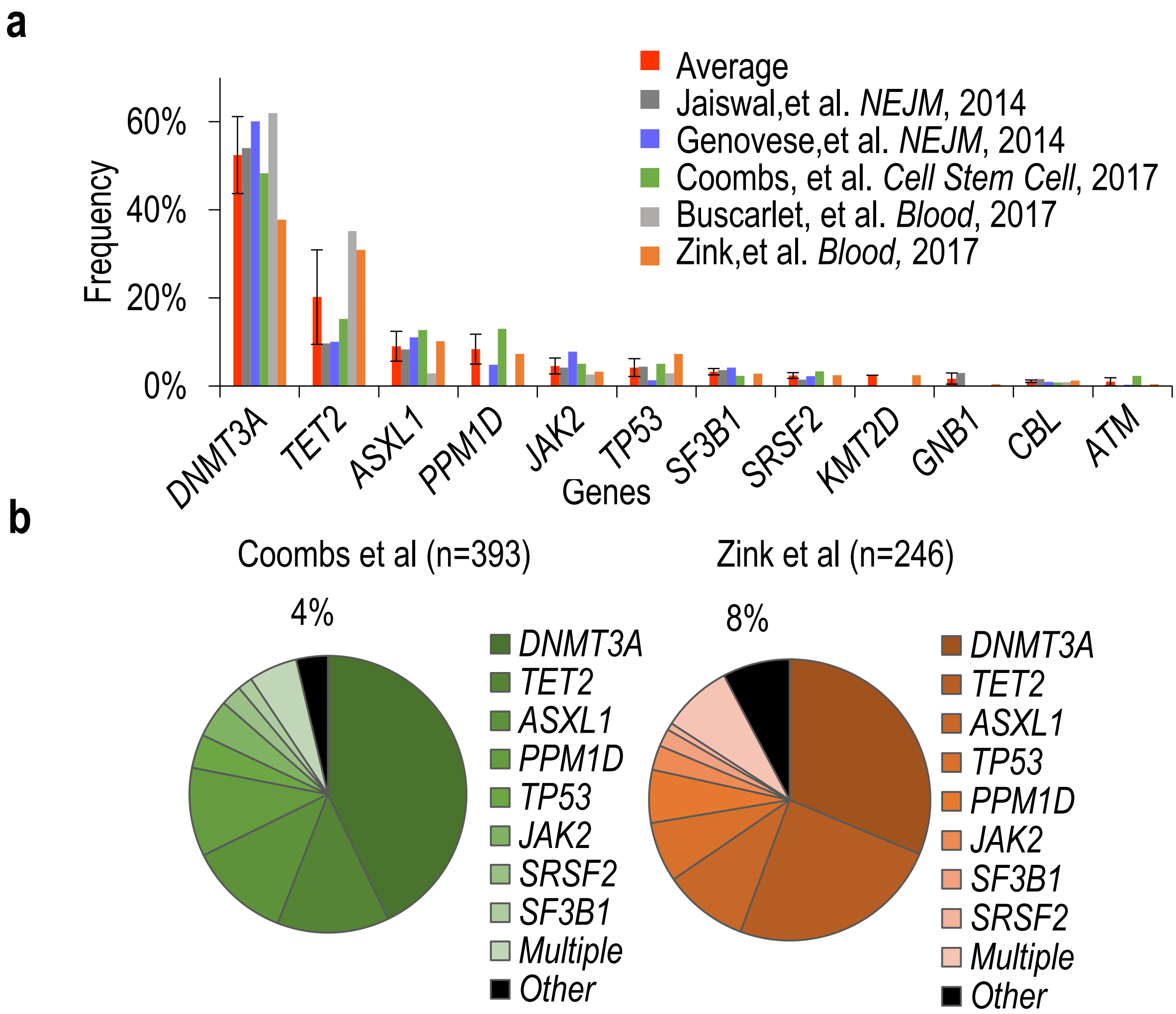

Supplemental Figure 18. Frequently mutated genes in CH

(a) Frequently mutated genes in CHIP meta-analysis[3-7]. Bar graphs show frequencies of mutations in CHIP from different cohorts. Red bars and error bar indicate mean frequencies and 1 standard deviation. (b) Fraction of mutation identified in top 8 genes in other 2 different CH cohorts. patients with multiple mutations in the 8 genes belong to the group called “Multiple”. Proportions of patients without them are also shown.

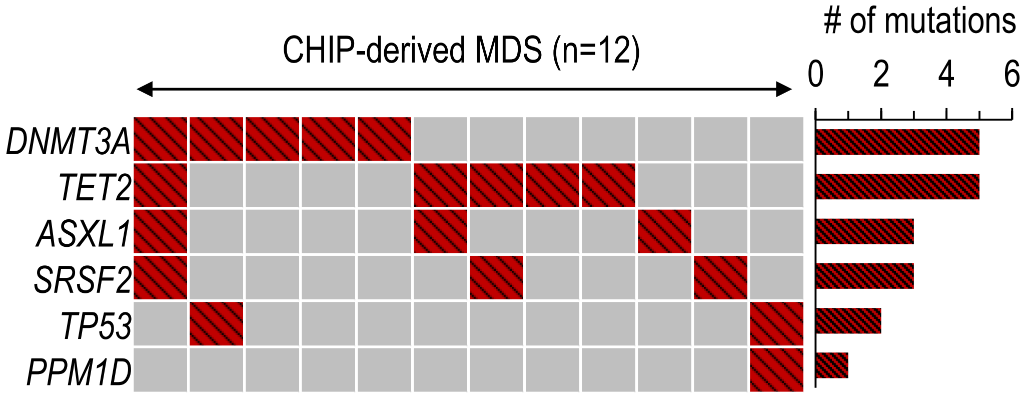

Supplemental Figure 19. Distribution of genetic mutations identified in CH-derived MDS

Distribution of mutations in CH-derived MDS patients from reported different cohorts. Side bar graph shows number of mutations in each gene.

Supplemental Table 1. Panel of 36 genes for targeted deep sequencing

ASXL1	*GATA2*	*NF1*	*SMC3*
BCOR	*GNB1*	*NPM1*	*SRSF2*
BCORL1	*IDH1*	*NRAS*	*STAG2*
CBL	*IDH2*	*PHF6*	*TET2*
CEBPA	*JAK2*	*PRPF8*	*TP53*
DNMT3A	*KDM6A*	*PTPN11*	*U2AF1*
ETV6	*KIT*	*RAD21*	*U2AF2*
EZH2	*KRAS*	*RUNX1*	*WT1*
FLT3	*MED12*	*SF3B1*	*ZRSR2*

Supplemental Table 2. Comparison of frequently mutated genes between WES and TS

TOP10	WES*	TS#
1	***TET2***	***TET2***
2	***SF3B1***	***SF3B1***
3	***SRSF2***	***ASXL1***
4	***ASXL1***	***SRSF2***
5	***TP53***	***DNMT3A***
6	***RUNX1***	***RUNX1***
7	***DNMT3A***	***STAG2***
8	***U2AF1***	***U2AF1***
9	***STAG2***	***TP53***
10	*EZH2*	*ZRSR2*

*: Whole exome sequencing cohort (n = 225), #: Targeted sequencing cohort (n = 1584)

Supplemental Table 3. Significant association with high risk phenotype for pairs of dominant and secondary mutations

Dominant mutations	Secondary mutations	P value for high risk	Q value for high risk	Odds ratio for high risk and 95% CI	(A) # of patients with both dominant and secondary mutations	(B) # of patients without dominant nor secondary mutations	# of patients for high risk in (A)	# of patients for low risk in (A)	# of patients for high risk in (B)	# of patients for low risk in (B)
TP53	*TP53*	0.001	0.008	13 (3.7-81)	19	1683	17	2	670	1013
RUNX1	*ASXL1*	0.001	0.008	5.3 (2.1-16)	22	1546	17	5	604	942
RUNX1	*STAG2*	0.001	0.008	11 (3.2-71)	17	1669	15	2	669	1000
EZH2	*ASXL1*	0.001	0.008	4.8 (2.0-13)	25	1559	19	6	622	937
SF3B1	*JAK2*	0.000	0.009	0.12 (0.03-0.34)	29	1411	3	26	697	714
SRSF2	*STAG2*	0.000	0.014	14 (4.1-88)	21	1584	19	2	639	945
U2AF1	*ASXL1*	0.004	0.023	2.9 (1.4-6.3)	32	1522	21	11	601	921
STAG2	*ASXL1*	0.006	0.026	4.2 (1.6-13)	19	1556	14	5	618	938
BCOR	*RUNX1*	0.015	0.054	13 (2.5-242)	10	1662	9	1	676	986
SF3B1	*DNMT3A*	0.013	0.055	0.28 (0.09-0.72)	23	1400	5	18	692	708
SRSF2	*ASXL1*	0.019	0.064	2.0 (1.1-3.6)	48	1483	27	21	580	903
RUNX1	*IDH2*	0.030	0.093	10 (1.8-190)	8	1721	7	1	702	1019

Supplemental Table 4. Association of gene mutations with response rate to hypomethylating therapy

Mutated genes*	P value*	OR (95%CI)*	NR, Mut(-)	NR, Mut(+)	R, Mut(-)	R, Mut(+)	P value†	OR (95%CI)†	CR(+), Mut(-)	CR(+), Mut(+)
TET2 -mut	**0.014**	2.4 (1.2-4.88)	92	20	44	23	**0.027**	2.63 (1.1-4.88)	21	12
ASXL1 -mut	**0.012**	0.32 (0.12-0.74)	82	30	60	7	**0.043**	0.27 (0.06-0.74)	30	3
TET2 -mut+*ASXL1* -WT	**0.003**	3.3 (1.5-7.52)	100	12	48	19	**0.008**	3.62 (1.38-7.52)	23	10
TET2 -mut+*ASXL1* -mut	0.762	0.83 (0.21-2.73)	104	8	63	4	0.829	0.84 (0.12-2.73)	31	2
TET2 -Dominant mut	**0.017**	2.57 (1.19-5.68)	98	14	49	18	**0.046**	2.62 (0.99-5.68)	24	9
TET2 -Secondary mut	0.156	1.83 (0.79-4.27)	99	13	54	13	0.166	2.05 (0.71-4.27)	26	7
ASXL1 -Dominant mut	0.097	0.38 (0.11-1.09)	96	16	63	4	0.441	0.6 (0.13-1.09)	30	3
ASXL1 -Secondary mut	0.089	0.33 (0.07-1.05)	98	14	64	3	**0.040**	Inf (Inf-0.88)	33	0
DNMT3A -mut	0.873	0.93 (0.37-2.21)	96	16	58	9	0.441	0.6 (0.13-2.21)	30	3
TP53 -mut	0.585	1.34 (0.46-3.77)	103	9	60	7	0.847	1.14 (0.24-3.77)	30	3
U2AF1 -mut	**0.047**	0.22 (0.03-0.8)	98	14	65	2	0.310	0.45 (0.07-0.8)	31	2

Abbreviation: NR, Nonresponders; R, Responders

* Mut vs others between Responders (n = 67) vs Nonresponders (n = 112)

† Mut vs others between Complete Reponders (CR, n = 33) vs. non-responsders (n = 112)

Supplemental Table 5. Reports for clonal hematopiesis (CH)

Paper	Total cases	Condition and races of cases	platform	Criteria of CH	Depth (Mean)	# of genes which related to hematologic malignancies	# of cases with CH (Frequency in total cases)	# of cases who developed hematologic malignancies after CH	# of cases with MDS after CH (Frequency in CH cases)	Frequently mutated genes in CH
[3]Genovese,et al. *NEJM* , 2014	12,380	Healthy (6,245), Schizophrenia (4,970), Bipolar disorder (1,165), All Swedish	WES	0.1 < VAFs	95	>11	308 (2.5%)	9	2 (2/308, 0.6%)	*DNMT3A, TET2, ASXL1, PPM1D, SF3B1, SRSF2, TP53,CBL, U2AF1, IDH2*
[4]Jaiswal,et al. *NEJM* , 2014	17,182	Type2 DM (15,801), Healthy (1,381)	WES	0.02 < VAFs	84	160	746 (4.3%)	5	2 (2/746, 0.3%)	*DNMT3A, TET2, ASXL1, TP53, JAK2, SF3B1, GNB1, CBL, SRSF2, GNAS,*
[5]Coombs, et al. *Cell Stem Cell* , 2017	8,810	Solid tumor,	TS	0.1 < VAFs*	419	35	393 (4.5%)*	7	4 (4/393, 1.0%)	*DNMT3A, TET2, PPM1D, ASXL1, TP53, SRSF2, SF3B1,ATM,CBL, JAK2, CHEK2*
[6]Buscarlet, et al. *Blood* , 2017	2,530	Healthy woman, French and Canadian	TS	0.04 < VAFs < 0.92	4,000	19	347 (13.7%)	NA	NA	*DNMT3A, TET2, ASXL1, TP53, JAK2, CBL, KRAS, NRAS, RUNX1, CEBPA, IDH2*
[7]Zink,et al. *Blood,* 2017	11,262	Healthy, Icelander	WGS	0.11 < VAFs <0.2	36	18	246 (2.2%)	9	4 (4/246, 1.6%)	*DNMT3A, TET2, ASXL1, TP53, PPM1D, CUX1, JAK2, SF3B1, SRSF2, KMT2D, CBL, BCOR*

* Clonal hemetopoiesis presumptive leukemia driver mutations (CH-PD)

Supplemental Table 6. Penetrance of genetic mutations from CH to CH-derived MDS

Genes	# of cases in CH derived MDS (n=12)	# of cases in CH (n=1,693)	Penetrance (CH to CH derived MDS)
DNMT3A	5 (42%)	888 (52%)	0.6%
TET2	5 (42%)	342 (20%)	1.5%
ASXL1	3 (25%)	153 (9%)	2.0%
SRSF2	3 (25%)	40 (2%)	7.5%
TP53	2 (17%)	71 (4%)	2.8%
PPM1D	1 (8%)	142 (8%)	0.7%
JAK2	0 (0%)	77 (5%)	0%
SF3B1	0 (0%)	55 (3%)	0%
Other	1 (8%)	74 (4%)	2.7%

Supplementary References

1. Makishima, H. et al. Dynamics of clonal evolution in myelodysplastic syndromes*. Nat Genet* 49, 204-212 (2017).

2. Yoshida, K. et al. Frequent pathway mutations of splicing machinery in myelodysplasia. *Nature* 478, 64-9 (2011).

3. Genovese, G. et al. Clonal hematopoiesis and blood-cancer risk inferred from blood DNA sequence. *N Engl J Med* 371, 2477-87 (2014).

4. Jaiswal, S. et al. Age-related clonal hematopoiesis associated with adverse outcomes. *N Engl J Med* 371, 2488-98 (2014).

5. Coombs, C.C. et al. Therapy-Related Clonal Hematopoiesis in Patients with Non-hematologic Cancers Is Common and Associated with Adverse Clinical Outcomes. *Cell Stem Cell* 21, 374-382.e4 (2017)

6. Buscarlet, M. et al. DNMT3A and TET2 dominate clonal hematopoiesis and demonstrate benign phenotypes and different genetic predispositions. *Blood* 130, 753-762 (2017)

7. Zink, F. et al. Clonal hematopoiesis, with and without candidate driver mutations, is common in the elderly. *Blood* 130, 742-752 (2017)
