# Supplementary material for: Invariant patterns of clonal succession determine specific clinical features of myelodysplastic syndromes
Source: Nat Commun. 2019 Nov 26;10:5386. doi: 10.1038/s41467-019-13001-y (PMC6879617; doi:10.1038/s41467-019-13001-y)
Supplement: Supplementary file 2 — Description of Additional Supplementary Files [file 41467_2019_13001_MOESM2_ESM.pdf]

Description of Additional Supplementary Files

File Name: Supplementary Data 1
Description: Significant association in frequent mutations/copy number alterations.

File Name: Supplementary Data 2
Description: Variants detected by targeted/whole exome sequencing and estimated clonal structure by PyClone.
