# Supplementary material for: Invariant patterns of clonal succession determine specific clinical features of myelodysplastic syndromes
Source: Nat Commun. 2019 Nov 26;10:5386. doi: 10.1038/s41467-019-13001-y (PMC6879617; doi:10.1038/s41467-019-13001-y)
Supplement: Supplementary file 5 — Reporting Summary [file 41467_2019_13001_MOESM5_ESM.pdf]

nature research

Corresponding author(s): Jaroslaw Maciejewski, Yasunobu Nagata

Last updated by author(s): Aug 14, 2019

# Reporting Summary

Nature Research wishes to improve the reproducibility of the work that we publish. This form provides structure for consistency and transparency in reporting. For further information on Nature Research policies, see Authors & Referees and the Editorial Policy Checklist.

## Statistics

For all statistical analyses, confirm that the following items are present in the figure legend, table legend, main text, or Methods section.

| n/a | Confirmed | |
|---|---|---|
| ☐ | ☒ | The exact sample size (*n*) for each experimental group/condition, given as a discrete number and unit of measurement |
| ☐ | ☒ | A statement on whether measurements were taken from distinct samples or whether the same sample was measured repeatedly |
| ☐ | ☒ | The statistical test(s) used AND whether they are one- or two-sided<br>*Only common tests should be described solely by name; describe more complex techniques in the Methods section.* |
| ☐ | ☒ | A description of all covariates tested |
| ☐ | ☒ | A description of any assumptions or corrections, such as tests of normality and adjustment for multiple comparisons |
| ☐ | ☒ | A full description of the statistical parameters including central tendency (e.g. means) or other basic estimates (e.g. regression coefficient) AND variation (e.g. standard deviation) or associated estimates of uncertainty (e.g. confidence intervals) |
| ☐ | ☒ | For null hypothesis testing, the test statistic (e.g. *F*, *t*, *r*) with confidence intervals, effect sizes, degrees of freedom and *P* value noted<br>*Give P values as exact values whenever suitable.* |
| ☐ | ☒ | For Bayesian analysis, information on the choice of priors and Markov chain Monte Carlo settings |
| ☐ | ☒ | For hierarchical and complex designs, identification of the appropriate level for tests and full reporting of outcomes |
| ☐ | ☒ | Estimates of effect sizes (e.g. Cohen's *d*, Pearson's *r*), indicating how they were calculated |

*Our web collection on statistics for biologists contains articles on many of the points above.*

## Software and code

Policy information about availability of computer code

| | |
|---|---|
| Data collection | Data was collected by electronic medical record system, Epic. |
| Data analysis | Data was analyzed using R (3.4.3), GraphPrism (v7) and Pyclone(ver 0.13.0). Further details can be found in the method section. |

For manuscripts utilizing custom algorithms or software that are central to the research but not yet described in published literature, software must be made available to editors/reviewers. We strongly encourage code deposition in a community repository (e.g. GitHub). See the Nature Research guidelines for submitting code & software for further information.

## Data

Policy information about availability of data

All manuscripts must include a data availability statement. This statement should provide the following information, where applicable:

- Accession codes, unique identifiers, or web links for publicly available datasets
- A list of figures that have associated raw data
- A description of any restrictions on data availability

The newly unreported targeted DNA sequencing data will be deposited in the database of Genotypes and Phenotypes (dbGAP) in NCBI, accession code will be available before publication.

# Field-specific reporting

Please select the one below that is the best fit for your research. If you are not sure, read the appropriate sections before making your selection.

☒ Life sciences ☐ Behavioural & social sciences ☐ Ecological, evolutionary & environmental sciences

For a reference copy of the document with all sections, see nature.com/documents/nr-reporting-summary-flat.pdf

# Life sciences study design

All studies must disclose on these points even when the disclosure is negative.

| | |
|---|---|
| Sample size | Our goal was to undertake the largest retrospective adult MDS data analysis to date. Statistical methods were not used to determine a sample size as more is always better. |
| Data exclusions | Cases with insufficient sequencing data were excluded. |
| Replication | Findings identified in our cohort of 1,809 MDS patients were compared to those reported in other papers. |
| Randomization | This is a retrospective analysis of patients not randomized to any treatment types. |
| Blinding | Blinding is not relevant as this is a retrospective data analysis |

# Reporting for specific materials, systems and methods

We require information from authors about some types of materials, experimental systems and methods used in many studies. Here, indicate whether each material, system or method listed is relevant to your study. If you are not sure if a list item applies to your research, read the appropriate section before selecting a response.

## Materials & experimental systems

| n/a | Involved in the study | |
|---|---|---|
| ☒ | ☐ | Antibodies |
| ☒ | ☐ | Eukaryotic cell lines |
| ☒ | ☐ | Palaeontology |
| ☒ | ☐ | Animals and other organisms |
| ☐ | ☒ | Human research participants |
| ☒ | ☐ | Clinical data |

## Methods

| n/a | Involved in the study | |
|---|---|---|
| ☒ | ☐ | ChIP-seq |
| ☒ | ☐ | Flow cytometry |
| ☒ | ☐ | MRI-based neuroimaging |

## Human research participants

Policy information about studies involving human research participants

| | |
|---|---|
| Population characteristics | Covariates studied include age, sex, and WHO disease classification. Given in Supplemental Table 1, for our 1,809 cases the median age was 71.8, the M/F ratio was 1.6, and there were 1,446 (80%) MDS, 212 (12%) MDS/MPN, and 151 (8%) secondary AML cases. Partitioning by risk, there were 1043 (60%) low-risk cases and 766 (40%) high risk cases. |
| Recruitment | Patients with MDS, MDS/MPN and related secondary AML were obtained after written informed consent, from Cleveland Clinic following the protocol (IRB-5024). Therapy-related MDS and AML were not included. |
| Ethics oversight | Our protocol was approved by our Cleveland Clinic's Institutional Review Board (protocol number; IRB-5024) |

Note that full information on the approval of the study protocol must also be provided in the manuscript.
